# Access or continuity: a zero sum game? A systematic review of the literature examining the relationship between access and continuity in primary healthcare

**DOI:** 10.1186/s12875-025-02860-8

**Published:** 2025-07-02

**Authors:** Mhorag Goff, Ali Hindi, Jonathan Hammond, Sally Jacobs

**Affiliations:** University of Manchester, Manchester, UK

**Keywords:** Primary healthcare, Access, Continuity of care, Systematic review

## Abstract

**Background:**

In recent years there has been a policy drive in the UK to improve patients’ access to appointments in primary care. However, the focus on timely access could undermine continuity of care. This paper aims to investigate how continuity of care and access to care are interrelated and their relative importance for patients and healthcare professionals.

**Methods:**

A systematic review was conducted using six academic databases (EMBASE, PubMed, Scopus, Web of Science, CINAHL and PsycINFO). Reference lists of included studies and Google Scholar were searched for additional papers. Included were peer-reviewed journal articles in English based on studies in primary care settings from any country, publication date and study design, based on data from any stakeholders. Conference abstracts, opinion papers, reports and literature reviews, studies in secondary or tertiary care or continuity *between* healthcare settings and studies about development of instruments to measure continuity of care or examining outcomes only were excluded. Fifty-six papers were identified for inclusion in the review. Studies presented differing perspectives on continuity and access, conceptualisations of access and continuity, and, measures used. We conducted thematic analysis of the literature and used Haggerty et al.’s (2003) conceptualization of continuity and Boyle et al.’s (2020) conceptualization of access to synthesise the data.

**Findings:**

Themes arising were: system-level, practice-level and patient-level factors that influence access and continuity of care, what is important to patients, and how providers can support access and continuity of care. We found that ‘choice of access’ has the strongest relationship with relational continuity, however, ‘physical access’, or the ability to get and ‘attend’ an appointment, supersedes other dimensions of access as necessary but not sufficient for continuity of care.

**Conclusions:**

Our synthesis provides evidence that experiencing continuity depend on the combination of patients’ demographic characteristics and health conditions, with situational circumstances, including characteristics of the health system and provider, which are more or less changeable. We propose a theoretical framing of the relationships between the dimensions of access and continuity. It can support policymakers and providers in understanding how to balance providing both access and continuity for patients.

**Supplementary Information:**

The online version contains supplementary material available at 10.1186/s12875-025-02860-8.

## Background

There has been a noted decline in continuity of care in UK general practice over the past two decades [1]. Yet continuity of care within primary healthcare, particularly in terms of the longitudinal relationship between the patient and their general practitioner (also known as a GP, family doctor or family physician), is recognised as one of its defining features [2]. It is particularly relevant to primary care in the UK where general practice is a ‘first responder’ provider [3] because the GP takes the lead role in treating and commissioning patient treatment. The conceptualisation of GPs as ‘family doctors’ indicates an expectation of a longitudinal relationship between doctor and patient across the patient’s life course [2, 4], where the care relationship between practitioner and patient often extends beyond specific episodes of illness or disease [5]. The importance of continuity of care in primary care has also been acknowledged internationally, with policy and organisational interventions aimed at optimising continuity of care in countries including the US, Canada, Rwanda, Namibia, Brazil, the Netherlands, Australia and Portugal [6].

A significant body of research has developed around continuity of care in different healthcare settings and for specific health conditions or groups of patients, particularly those with chronic conditions such as diabetes [7, 8], and multiple comorbidities [9]. A subset of this literature has sought to determine the relationships between continuity of care and a range of different patient and system-related outcomes [8]. This research has established strong evidence for the importance of continuity of care in a number of respects, including for patient mortality [8, 10], health outcomes and patient satisfaction [11], in addition to health system efficiency [7].

Nevertheless, in response to increasing patient demand and an overstretched general practice workforce [12], UK primary healthcare policy has prioritised improving access to services for patients [13, 14]. Instigated in 2002-2003 [15] the ‘Advanced Access’ initiative incentivised practices to provide patients with appointments on the day of their choosing [16]. However, within many practices, this reduced the availability of ‘routine’ advance appointments by prioritising same day consultations [15]. In England, policy initiatives have also focused on increasing the provision of out-of-hours services in general practice. It has been argued that the increased focus on timely access has been at the expense of continuity of care [13, 17, 18], although the notion of a trade-off has also been characterised as unproductive and misleading [8, 19]. In response there have been increasing calls from primary care professionals and their professional bodies for greater emphasis on continuity of care and for measures to be taken to mitigate its loss [2].

Continuity of care is traditionally (often implicitly) framed as the ongoing relationship between a patient and the doctor treating them, and much of the existing evidence of the benefits of continuity of care tends to adopt this definition. However, from a theoretical perspective, there has been a proliferation of definitions and conceptualisations of continuity of care [20]. Initially these focussed narrowly on patients having a personal care provider e.g. [21], while a later, broader definition of continuity highlighted the importance of patients receiving coordinated and uninterrupted care e.g. [22].

More recently, evidence-based definitions of continuity of care have emerged, including Haggerty et al.’s [5] widely-used conceptual framework, which is grounded in a multi-stage evidence synthesis. It comprises three types of continuity of care: informational continuity, management continuity and relational continuity. Relational continuity is the longitudinal relationship between a patient and an individual clinician/doctor over time; management continuity is the coordination of a patient’s treatment across multiple clinicians, healthcare settings and over time for an episode of disease; and informational continuity is the sharing of significant patient information to enable care to be handed seamlessly between healthcare professionals. Haggerty et al.’s conceptualisation will be used in this paper to frame our analysis of the literature.

Similarly, a number of definitions and conceptualisations of access to care have been used in the literature [14, 23], and various measures quantifying access have been used in policy and research [14]. The notion of access to care can include acceptability, timely access, convenience, physical access and choice [24], encompassing notions of potential as well as realised access [25] and the interplay of service provision and patient agency [23].

Levesque et al. [23] conceptualised access at the interface of health systems and populations via synthesis of the existing literature. Levesque defines access to healthcare as “the opportunity to reach and obtain appropriate health care services in situations of perceived need for care” [23](p4). Levesque’s framework specifically focuses on the accessibility of providers, organisations, institutions and systems, and the abilities of populations, communities, households and individuals to engage with healthcare organisations. The framework offers five dimensions of accessibility of services and five corresponding abilities of people to interact with the dimensions of accessibility to generate access. These factors include structural characteristics of health systems determined by the national and/or regional healthcare system that shape providers’ ability to provide effective care for particular patients, and cultural factors, such as providers’ access policies and staffing levels. Levesque’s framework was further developed to centre on the notion of patient-centred access as ‘fit’ between the needs and abilities of people in the population and the abilities and capacities of people in the healthcare workforce [26].

Boyle et al. [14] proposed a framework for measuring access in general practice that combines key aspects of access based on a rapid review of data from the English NHS GP Patient Survey [27]. It includes the following dimensions: “physical access” (availability of GPs, proximity, design of premises, telephone access, home visits, electronic access); “timely access” (appointment and booking hours, out-of-hours care, waiting times for appointments and for prescriptions); and “choice” (choice of practice, choice of professional). We use Boyle et al.’s conceptualisation of access to analyse the review data.

Despite the range of conceptualisations, the empirical literature does not tend to differentiate between the different dimensions of access and continuity, excepting literature that addresses access policies. In addition, most of the literature to date has treated access and continuity as separate entities [13]. While much has been made of the apparent trade-off between access to care and continuity of care [28, 29, 30], empirical evidence for the relationship between these elements of primary care has been sparse and previous systematic reviews of evidence about continuity of care and about access to care have not addressed the relationship between them.

Our paper aims to address this gap by synthesising the research evidence that addresses both access to and continuity of care. In this systematic review we address the question of how continuity of care and access to care are interrelated, how provider organisations may support access and continuity, what is most important to patients and healthcare professionals, and under what circumstances. Drawing on our findings we then propose a new theoretical framework which incorporates the relationships between access and continuity.

## Methods

Our paper aims to synthesise the research evidence about access to and continuity of care to determine how they are interrelated.

### Study design

A systematic review of the international literature with narrative synthesis was undertaken, providing a descriptive account of both qualitative and quantitative research findings [31].

### Information sources and search

Six academic literature databases (EMBASE, PubMed, Scopus, Web of Science, The Cumulative Index to Nursing and Allied Health Literature (CINAHL) and PsycINFO) were searched for relevant literature. Search terms were developed by all authors and reviewed by a librarian (Table 1). The specific search strategies for each database are provided in Appendix 1. References of included studies and Google Scholar were used to search for additional literature. The original searches took place from March to May 2021. The searches were then rerun in May 2023 to capture the most recently published evidence.

**Table 1:** Search terms used for the review

Concept	Search terms
Access	“Access to health care” OR “access to healthcare” OR “extended access”
**AND** Continuity	Continuity OR “continuity of care” OR “continuity of patient care” OR “care continuity” OR coordination or co-ordination OR “patient care” OR “relational continuity”
**AND** Primary care	“primary care” OR “primary medical care” OR “general practice” OR “general practitioner” OR “family physician” OR “family doctor” OR “family medicine” OR GP or FP

### Data screening

Titles and abstracts were initially screened against the inclusion/exclusion criteria by AH (Table 2). Subsequent screening involved full-text application of the inclusion/exclusion criteria by AH and MG. All authors were consulted where queries arose.

**Table 2:** Inclusion and exclusion criteria

Inclusion Criteria	Exclusion Criteria
**Setting**: Primary care	**Setting**: secondary care; tertiary care
**Location**: All countries	
**Design/Study type**: Any	
**Publication type**: Peer reviewed journal papers	**Publication type**: Conference abstracts; opinion papers; reports; literature reviews
**Language**: English **Publication date**: Any	**Language**: languages other than English
**Participants**: Any stakeholders. Any patient group, including entire populations or groups of patients with a specific disease or other feature.	
**Focus of study**: - Perceptions/experiences of both access and continuity in primary care - Patient preferred practice attributes when utilizing primary care services (where access and continuity of care are covered) - Views on trade-offs between access and continuity - Patient satisfaction with primary care services (where access and continuity are covered)	**Focus of study**: - Either continuity of care or access to services without any examination of the other - Only the association between access/continuity and health outcomes - Care-coordination/continuity between primary care and other settings (e.g. hospital) - Development and/or validation of instruments measuring continuity of care

### Data extraction, analysis and synthesis

Data from all included articles were extracted by AH and MG and then reviewed with SJ. The data extracted were collated in tabular form summarizing study characteristics (Appendix 1): author(s), year of publication, country, study aim, study design, number of participants, how access and/or continuity were measured, relevant findings and themes. Themes were identified by AH using the following steps. Relevant extracts from included studies were entered in NVivo and independently coded [32]. Codes from included studies that demonstrated commonalities were combined under initial broad themes. Initial themes were further analysed to interpret underlying meanings and closely examined and redefined through team meetings. Final themes and their interpretations were presented to the wider team in a meeting to obtain structured feedback and validate the common themes. A narrative approach was taken to reporting study findings within each of the themes and sub-themes.

In addition to thematic analysis of the literature, our synthesis used Haggerty et al.’s [5] conceptualization of continuity, comprising relational, management and informational continuity. We also used Boyle et al.’s [14] conceptualization of access as comprising physical access, timely access and choice. These two conceptual frameworks for continuity and access were chosen for their relative simplicity in terms of numbers of dimensions (compared with other conceptualisations). This made them more applicable to heterogeneous data, in which access and/or continuity of care were not necessarily defined or theorised. These concepts were used as a means of structuring the data and provide a basis for building our theoretical case for the relationship between access and continuity.

### Findings

**Fig. 1:**
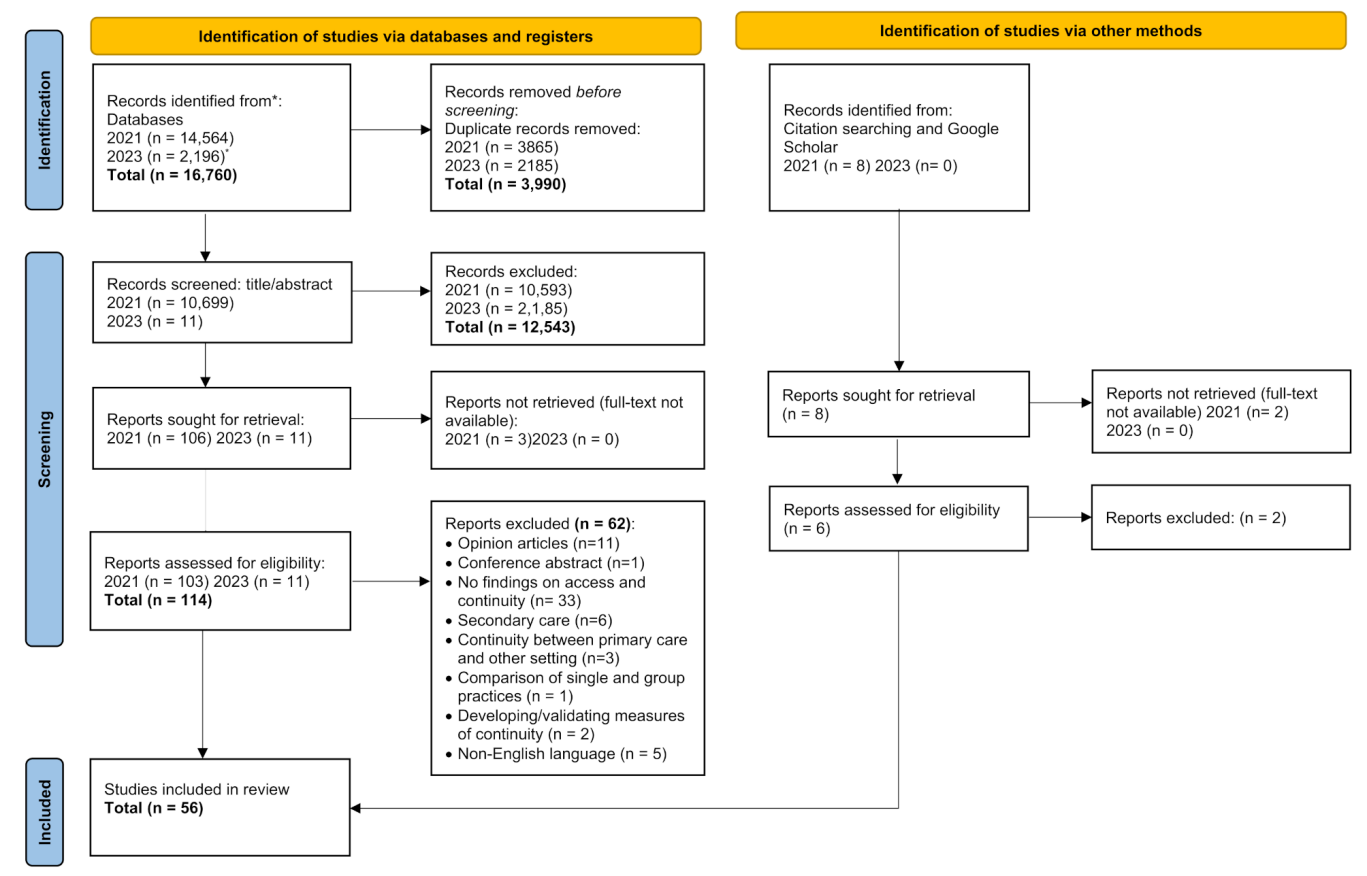
Literature search process using PRISMA

After removing duplicates, a total of 10,699 papers from the original search were subjected to initial screening. This process was repeated in May 2023 to bring the evidence up to date as shown in Fig. 1. Following initial screening, 106 papers were sought for full-text reading in 2021 and 11 further papers in 2023. Additionally, a search of citations within these papers as well as a Google Scholar search yielded eight papers in 2021 and no additional papers in 2023. The full text of five of these were unavailable, meaning 120 papers in total were assessed for eligibility from both search methods across 2021 and 2023 combined. 64 papers were excluded as not meeting eligibility criteria. The remaining 56 papers were included in the review.

### Study characteristics

The majority of papers reported studies from the UK (*n* = 18) [11, 16, 28, 30, 33–46] followed by Canada (*n* = 12) [18, 29, 47–56] and the US (*n* = 9) [57–65], with the remainder being from Portugal [66, 67], Poland [68], the Netherlands [69–71], Norway [72], Sweden [73], Switzerland [74], Germany [75], Finland [76], Australia [77, 78], New Zealand [79], France [80], Singapore [81], Lebanon [82] Brazil [83] and China [84].

Thirty-seven studies explored the views of patients [11, 16, 29, 30, 34, 35, 37–43, 47, 48, 50, 51, 54, 56–59, 61, 64, 66–68, 70, 71, 74, 75–77, 79, 82–84], five explored the views of GPs [36, 62, 72, 80, 81], other papers addressed the perspectives of: both patients’ and GPs [33, 36, 52, 60], patients and multiple primary care healthcare professionals [73], patients and carers [78], service users and providers [46, 53], GPs and practice nurses [65, 69], clinicians, academics and NHS decision-makers [49], administrative staff [45], and one explored the views of reception staff [28].

Most studies used self-report questionnaires (including discrete choice experiments and Delphi methods) (*n* = 29), followed by semi-structured interviews (*n* = 10). Nine studies used more than one method of data collection. The remaining papers involved retrospective analysis of administrative data (*n* = 4), focus groups (*n* = 3), nominal group techniques (*n* = 1), one quality improvement intervention and one before and after simulated patient study.

In terms of focus, 32 papers explored patient and/or GP preferences for access and continuity of care, some of which considered relative preferences or trade-offs. Ten papers explored the experiences of access to and/or continuity of care of particular patient populations [39, 42, 44, 47, 53, 70, 73, 78, 79], and nine papers examined the influence of organizational factors in general practice on access and/or continuity [11, 28, 41, 50, 52, 60–62, 72]. Five papers examined the impact of specific organisational interventions to improve access and/or continuity: three studied the use of Advanced Access booking systems and their relative impacts on access and continuity [16, 30, 34], and one study interventions to improve both access and continuity [63]. Patient, provider and system-level factors that influence access and continuity are outlined in Table 3.

**Table 3:** Factors that influence access and continuity

Health system characteristics	Provider characteristics	Patient characteristics
Personal list or non-list-based	Numbers of GPs	Gender
Free at point of care or fee for service	Part-time or full-time GPs	Educational attainment
	Practice/provider population size	Ethnicity
	Geographical location	Chronic condition
	Access policy: • Out of hours availability • Telephone availability • Remote or in person appointments • Prioritisation of urgent or routine appointments	Multimorbidity

[Appendix 1: Summary of included studies].

### Narrative synthesis

Five themes emerged from the analyisis of existing literature: “system-level factors that influence access and continuity of care”; “practice-level factors that influence access and continuity of care”; “patient-level factors that influence access and continuity of care”, “what is important to patients” and “how can providers support access and continuity of care”. Each theme will be described below with a particular focus on what can be learned about the relationship between access and continuity of primary healthcare.

### System-level factors that influence access and continuity of care

This theme addresses how national and regional access policies and models of healthcare delivery influence access and continuity in general practice.

#### Healthcare delivery models

Healthcare systems may require patients to register with a single provider or, conversely, allow them to visit multiple providers. Similarly, systems may be based on GPs having personal lists of patients they take responsibility for, or allow patients to visit any GP at a given provider, or a mixture of both. Patients who are registered with a specific provider are said to be ‘attached’.

Where GPs maintain their own patient lists this supports relational continuity [11, 72]. Conversely, in healthcare systems where patients do not have to register with a provider, or in which GPs do not have personal lists, there may be less relational continuity as a consequence of patients seeing multiple GPs [85]. Personal lists have been found to improve physical access in terms of the ease of getting to the general practice, and of timely access to appointments [11]. Providing a named GP for each patient supported relational continuity in Portugal by ensuring consistency in the clinician patients consult with [67]. Recent health system reforms in the UK focused on scaling up care delivery across multiple collaborating providers mean that many UK general practices now use shared lists, meaning relational continuity is not guaranteed [86]. However, introducing named accountable GPs for patients may not in itself translate into better continuity of care when assessed by the proportion of contacts with the named GP [87].

In Canada, many patients remain ‘unattached’ to a provider’s list i.e. unregistered with a care provider [54, 55]. By definition, ‘unattached’ patients have poorer access to care than those attached to a primary care provider, and instead have to use walk-in centres or emergency departments [54]. Unattached patients’ lack of continuity hindered identification of emergent health needs and meant chronic conditions had to be largely self-managed, which risked poorer health outcomes. When patients became attached to a primary care provider their use of primary care increased, suggesting an improvement in access to and continuity of primary care [48, 54].

Whether healthcare is insurance-based or tax-based can also affect access and continuity of care. In the US, health insurance coverage influences both access and continuity of care such that uninsured patients reported having poorer physical access to care and less relational continuity with GPs [57].

#### Access policies

Long waiting times for appointments, poor physical access and lack of available care providers are commonly identified barriers to continuity of care for patients [44, 47, 49, 50, 57, 67]. In addition, appointment booking policies that prevented patients from booking advance appointments with a chosen GP undermined continuity of care [35, 67, 80].

The literature indicated contradictory findings about the ‘Advanced Access’ policy in English general practice, which aimed to offer doctor’s appointments to patients within 48 hours to improve the timeliness of access [14, 28]. Appointment booking systems in 3 English practices were compared; one had implemented Advanced Access, and the other two practices had not. The study compared speed of access to an appointment with whether patients had seen their preferred GP, assessed using a questionnaire. Advanced Access restrictions placed on booking ‘routine’ advance appointments were found to limit patients’ ability to see the GP of their choice, particularly for those with chronic conditions, despite providing faster access to appointments [30]. Conversely, a simulated patient study that compared 24 English practices that had implemented the Advanced Access policy with 24 that had not, found that patients were no more or less likely to get an appointment with their preferred GP [16]. Researchers had anonymously posed as patients to call practices to make an appointment for a non-urgent issue to assess timeliness, and a sample of anonymised patient records were assessed for continuity of care on the basis of the proportion of consultations with the same GP.

How the included studies assessed continuity differed in scale, which may be the source of their divergent findings on continuity. However, this may also point to the need to further investigate more subtle aspects of patients’ preferences, including where patients may not have a preferred GP. Reception staff were also shown to have a critical role in supporting (or undermining) providers’ access policies. In practices in England that had implemented a rapid access policy, reception staff reported placing less emphasis on patients seeing the same healthcare professional, interrupting episodic or longer-term relational continuity between a patient and clinician. However, receptionists in practices with access policies that facilitated booking patients with their named GP were less inclined to offer patients an appointment with another GP even when the named GP was unavailable on the same day [28].

There is some evidence that pre-bookable, shorter length ‘review’ appointments booked with the same GP or Advanced Nurse Practitioner as the patient’s initial appointment can improve relational continuity [45]. In addition to ensuring patients saw the same clinician across care episodes, the intervention improved access by making review appointments shorter, thereby increasing the availability of all appointment types [45].

This evidence suggests that facilitation of ‘routine’ appointments for chronic care or follow up is important in enabling relational continuity, whereas initial and/or acute or ‘urgent’ appointments are associated with interventions that prioritise timely (quick) access.

### Practice-level factors that influence access and continuity of care

This theme describes research evidence related to general practice characteristics including staffing levels (clinical hours, full or part-time GP status, and GP numbers), practice population size and organisational culture influence access and continuity of care, and related dimensions including patient satisfaction.

#### Staffing levels and availability

Two distinct elements, namely individual GPs’ clinical hours (related to full or part-time working) and the numbers of GPs in a patient’s practice, had subtly different effects on relational continuity and on access.

The literature suggests that increasing GPs’ weekly clinical working hours increased both timely access and relational continuity for patients [60]. However, fewer clinical hours worked per week were associated with greater patient satisfaction, although reasons for this were not provided [60]. Similarly, patients of US Veterans Health Administration clinics with part-time GPs were less able to get same day appointments with their preferred GP than those whose preferred GP worked full-time [61]. Another framing of continuity, used in a Norwegian study, extended beyond patient consultations with their preferred GP to include patients contacting GPs with queries outside of consultations [72]. This means that clinical hours may matter for continuity not only because of their impact on formal appointment availability with a chosen GP but on broader availability of that GP.

Providers employing more GPs offered more timely access but less relational continuity to patients [52]. However, providers’ access policies, including whether the practice offered extended opening hours and the proportion of scheduled appointments available compared to walk-in appointments, mediated these effects.

#### Provider population size

As the size of the provider’s patient population increases, patient satisfaction with relational continuity, framed as the ability to see their preferred GP, and timely access, framed as ease of getting appointments with doctors, both decrease [11]. English general practices that had grown in population size between 2013 and 2018 had a greater fall in positive patient responses to relational continuity and to a lesser extent physical and timely access (i.e. ease of getting an appointment), compared to practices that had stayed the same size [41].

#### Organisational culture

A single study examined the relationship between organisational culture and patients’ experiences of access and continuity across 41 sites of a large US primary care medical provider [62]. It found that a developmental organisational culture, emphasizing external expansion and entrepreneurialism, and hierarchical culture reflecting internal stability and uniformity were associated both with less relational continuity (in terms of waiting times for routine appointments, the proportion of contacts with the named GP and satisfaction with care) and less timely access to care (in terms of longer waiting times for appointments) than primary care organisations with a group-oriented culture, which emphasizes trust and affiliation among members [62].

#### Changes to healthcare delivery

A qualitative study with patients in the Netherlands during the Covid-19 pandemic found that they had poorer experiences in terms of reduced accessibility to and continuity of GP care during a period of increased use of remote consulting. Experiences included feeling unwelcome, chronic care being postponed by the GP, and for Covid-related care, seeing unfamiliar doctors owing to care being segregated [71].

Larger scale healthcare provision through federated models, including Primary Care Networks in the English NHS, have implications for informational continuity. Healthcare professionals reported difficulties in accessing patients’ medical records from other providers within their networks where they lacked interoperable electronic medical record systems, which undermined informational continuity [80, 81].

### Patient-level factors that influence access and continuity of care

Several studies in this review highlight the influence of sociodemographic factors on patients’ experiences of, and preferences for access and continuity of care [39, 57, 77, 79]. Relational continuity was valued more by female patients [77, 82] and patients with lower educational attainment [82], and was more highly valued as the patient’s age increases [33].

Patient ethnicity was also shown to have a bearing upon their experiences of access and continuity. A US survey study found that Hispanic and Black non-Hispanic respondents faced significantly greater access barriers compared with the White non-Hispanic patients [57]. Barriers to access included patients’ inability to travel to the primary care provider, costs of primary health care, the availability of after-hours appointments, and appointment and ‘in-office’ waiting times. Patients living in poverty faced substantially greater barriers to accessing care than those living above the poverty line [57]. These barriers to timely and physical access led to lower levels of relational continuity among Hispanic and Black patients.

Where patients lived was relevant to access and continuity for some patients, particularly in sparsely populated rural areas where patients may be restricted to practices nearby [72]. Whether patients live in more urban or more rural areas may also influence patients’ expectations of access. Patients from larger cities have higher expectations of physical access to care, such as longer opening hours, and telephone access to the GP, compared to those living in rural areas [68].

Finally, for patients with chronic conditions, difficulties getting an appointment with the same GP were less pronounced for those with diabetes alone compared with patients with diabetes alongside other co-morbidities [39]. This highlighted that greater need to use primary care could make experiencing such challenges more likely.

### What elements of access and continuity are important to patients?

This theme considers the relative importance of different dimensions of access and continuity of care to different patients and patient cohorts under differing circumstances.

#### Access

In terms of access, shorter waiting times for appointments [18, 58] (timely access), greater availability of out-of-hours care [50, 51, 57], the option to have telephone consultations [50], longer hours of telephone access to healthcare professionals [69] (physical access), and adequate staffing levels in practices [62], were found by to promote better patient access to care according to patients.

#### Relational continuity

In terms of relational continuity, patients prefer a GP who they trust and who shows empathy [42]. Consistency of consulting with any given GP is no guarantee of relational continuity because it does not necessarily entail a good relationship with or the trust in the clinician that is necessary to relational continuity [66]. There can be downsides to relational continuity for both patients and clinicians, for example, if GPs overlook possible diagnoses with a familiar patient [36]. Patients who may have experienced discrimination in care can also experience challenges in getting relational continuity because of this need for a trusting relationship. In a qualitative New Zealand study Maori patients perceived that barriers to getting good quality and non-discriminatory care were reduced when they had relational continuity with a (usually non-Maori) GP whom they trusted [79]. Patients also want to know that they can contact their GP [73], and knowing how to do so increased the strength of relational continuity [73].

#### Informational and management continuity

Patients considered electronic medical records central to their care, enabling them to be seen by any GP or healthcare professional, rather than only one who knows them [47, 59, 67]. Patients want reliable information sharing about them between healthcare professionals to avoid them having to repeat the same information or act as information brokers [47]. Consistent health messages from healthcare professionals were considered an important indication of reliable information sharing [42, 78], and conversely receiving inconsistent advice, which could indicate a lack of informational continuity, was disconcerting for patients [42].

Informational continuity mattered less among more pro-active and less vulnerable patients [70]. Relational continuity was considered less important by patients if informational continuity was effective [67]. In this respect, part-time GPs were perceived to have no less knowledge of their patients’ significant medical history [61]. Patients want to be reassured that, with or without relational continuity, they are being cared for by healthcare professionals who have an understanding of their medical history irrespective of whether they have seen them before [40, 42].

While little attention has been paid to patients’ perspectives on management continuity, a study of primary care patients in China found they prioritised their doctor taking follow up actions on their behalf, second to the care priority of seeing the same doctor [84].

Differences in the priorities of patients in different countries highlighted above reflect the influence of system-level factors (whether the system is list-based, fee for service etc.), provider and patient characteristics, such as where patients live and the level of provision in their area.

#### Which is prioritised, access or continuity, by which patients and under what circumstances?

Rather than prioritising either timely access or relational continuity, several studies indicate that patients want *both* timely (quick) access to care and relational continuity [29, 56, 67, 88]. Moreover, more timely and better physical access to care, and better relational continuity of care, have been shown to be predictors of higher patient satisfaction with care [11, 51, 60, 64, 83]. Conversely, while patients in Swiss general practice considered relational and informational continuity to be more important than timely and physical access [74], in Poland, timely and physical access to general practice were perceived to be of greater importance by patients [68]. A survey of patients in China found that physical access in terms of services geographically close to patients was a priority [84].

Patients’ preferences for timely (quicker) access over relational continuity depended on their perception of the urgency of their health issue [59]. Under such circumstances patients were willing to speak to any clinician [35] and requesting to see specific a GP reduced timely access [51]. However, even for acute issues, speaking to a familiar clinician was preferable to patients because it made it quicker for them to explain a health issue to the GP, and could help patients communicate effectively when they might not know what questions to ask [35, 88]. The evidence indicates that patients and GPs concur about the importance of a personal patient-GP relationship for the care of complex and chronic conditions [33, 36, 37, 74, 85]. These patients are willing to wait longer for an appointment with their personal or preferred GP for regular check-ups or for the sudden onset of a new condition, whereas quicker access is valued for acute problems by all patients [29, 34, 37, 38, 64]. Patients with chronic conditions want to be cared for by healthcare professionals who understand their medical history, irrespective of whether they have seen them before [40, 42].

Timely access, relational continuity and coordinated care (relating to management continuity) were all high priorities for patients with chronic conditions [78] in addition to informational continuity in terms of their GP knowing their medical history so they are able to detect changes in their health status [73]. Patients with mental health conditions preferred to have consultations with a GP they knew and trusted [44].

### How can providers support access and continuity?

The literature suggests that certain approaches to healthcare provision can help to preserve aspects of access and continuity of care. These approaches include providers adjusting their access policies, team-based approaches to care and improvements to informational continuity.

#### Team-based approaches

Team-based approaches to providing continuity involve small teams of named healthcare professionals, rather than a single clinician, taking charge of providing care to a patient [63]. It is reported by healthcare professionals as strengthening care coordination (thereby management continuity) when the patient’s preferred GP is not available [47, 69, 78]. GPs and nurses perceived that working with other healthcare professionals to provide individualised care for patients with multimorbidities could help them detect additional problems [69].

#### Adjusting access policies

An intervention in US general practice that tested a team-based approach was reported to have helped to optimise patients’ access to the same group of clinicians, benefitting relational continuity as a consequence [63]. It did so by offering patients the first available appointment with their usual GP or one from the same team, monitoring supply and demand for different appointment types, allowing different appointment types to be booked into the clinical schedule, reducing demand by dealing with as many issues as possible in a single visit, and extending intervals between return visits where possible [63].

#### Enhancing informational continuity

Informational continuity via shared electronic care records supports coordinated care for patients across different providers within the healthcare system [89], making it important to management continuity. Effective documentation in medical records that are accessible to all clinicians within the provider team is essential for collaborative care and providing healthcare professionals with sufficient information to provide care [69].

## Discussion

This literature review identifies a range of factors, at health system, provider and patient levels that influence provision of access and continuity of care in primary care and how they are experienced by patients. Our synthesis provides evidence that the dimensions of access and continuity important to patient care depend on a combination of patients’ demographic characteristics and health conditions, and situational factors including characteristics of the health system and provider, which may be more or less changeable. Moreover, our findings indicate that the relationship between access and continuity in primary care is not a straightforward zero sum game. We therefore propose a theoretical framework that accounts for the differing relationships between the dimensions of access and continuity suggested by the literature.

### Theorising the relationship between access and continuity in primary care

**Fig. 2:**
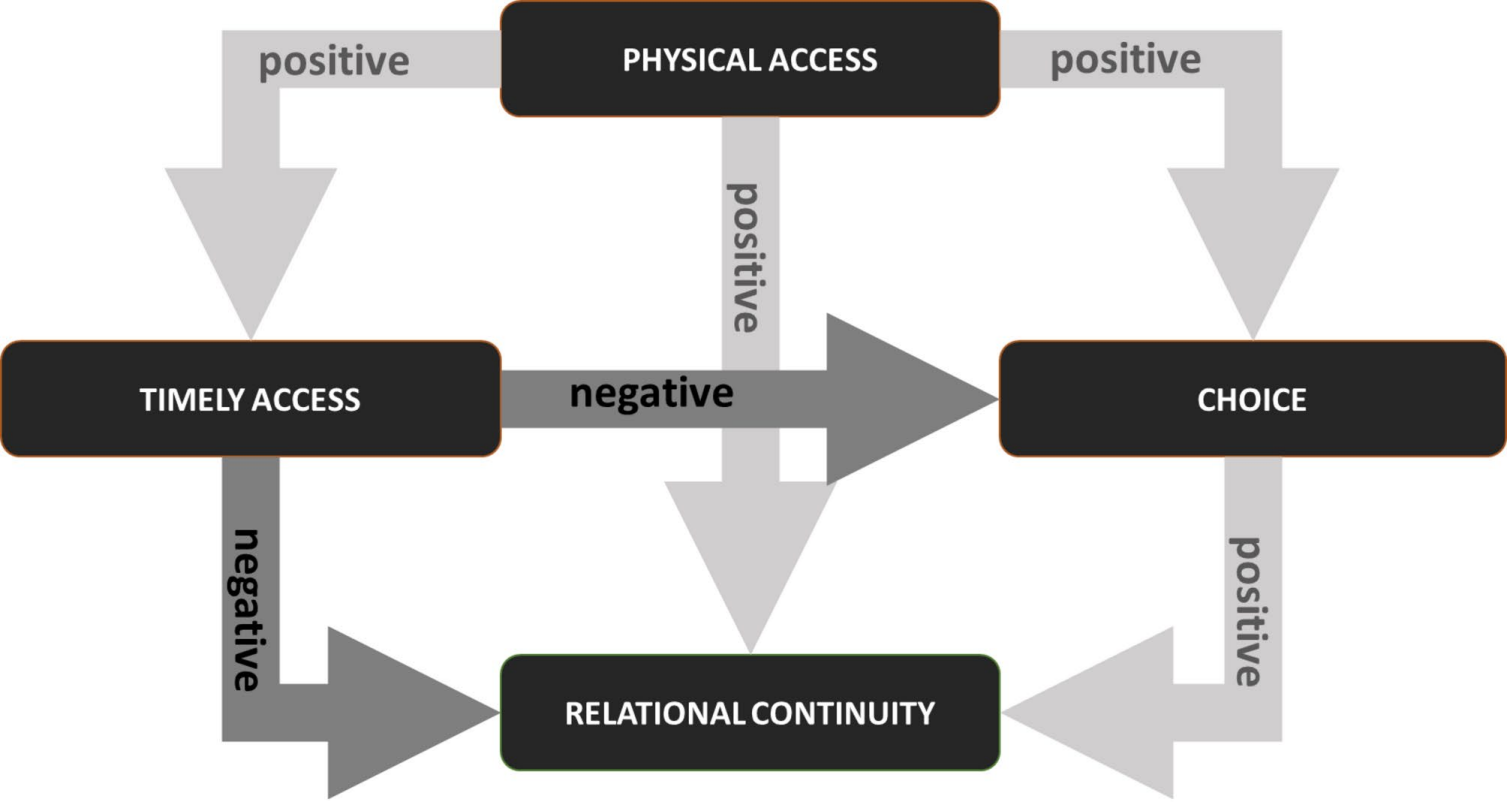
Relationships between dimensions of access and continuity of care

The literature reviewed in this paper allows us to examine the relationship between access and continuity across their respective dimensions, with evidence for relationships between the dimensions of access (*physical access*,* timely access and choice*) and *relational* continuity in the main. We capture these relationships in Fig. 2.

Although the interplay between access and continuity was not generally explicitly examined within the papers themselves, there was evidence of practices taking steps to preserve relational continuity through their access policies [28, 44, 51, 53]. As noted in the findings, practices’ access policies, including NHS England’s Advanced Access initiative [15], may undermine both choice and relational continiuity by prioritising timely access with *any* clinician over booking routine appointments with a chosen clinician. Conversely, access policies may bypass the need for patients to actively exercise choice in relation to which healthcare professional they see where practices themselves ensure that patients are able to always see the same clinician. This means that it is possible to have relational continuity without choice. However, without choice there is unlikely to be relational continuity where access policies rely on patients requesting an appointment with a specific GP.

In addition, patients may be unable, in practical terms, to choose a provider because they are in a rural area with fewer providers nearby, because of cost or because they have mobility or transport issues. Their preferred GP may only be available at certain times, for example, during office hours but not out of hours, or because they work part-time, therefore the scope for choice and for relational continuity may vary. In this way the choice dimension of access supports relational continuity for patients but does not guarantee it.

Our model suggests that although physical access is a pre-requisite to continuity of care, the ‘choice’ dimension of access dominates the relationship between access to and continuity of care. This is because patients having a choice of which clinician to consult, either within a given practice or from among a range of practices (depending on the health system), enables them to request an appointment with their preferred GP, facilitating relational continuity. However, choice is neither necessary nor sufficient for continuity of care.

Unlike choice, which acts as an enabler of relational continuity, timely access may hinder relational continuity, often through restrictions to choice, putting these dimensions in opposition in relation to their influence on relational continuity. This explains why choice of clinician ‘on paper’ may not translate to choice in practice because, for example, appointment times are inconvenient for some patients or there is a system-wide policy of prioritising same day appointments. Trade-offs by patients are apparent when they need to balance their need for timely access and choice to see a preferred clinician [29, 64].

As we chose to use Haggerty et al.’s [5] and Boyle et al.’s [14] conceptualisations of continuity and access respectively, we need to consider our analysis in the context of other theoretical conceptualisations that we might have used. While Levesque et al. [23] focus on access alone, our findings for both access and continuity reflect their recognition of a complex interplay between patients seeking care and the characteristics of the healthcare organisation. This is reflected in ‘good’ continuity of care for patients being dependent on the unique configuration of their own and the provider’s characteristics, which determines the ‘fit’ between them. It is the quality of this ‘fit’ between access and continuity that continuity is highly dependent on.

This review also highlights relationships between a range of dimensions of healthcare provision (e.g. staffing levels, patient population size and rurality), and patient characteristics, with access and continuity of care. These findings suggest that the relationship between timely access and choice is mediated by the patient-specific combination of characteristics at system, practice and patient levels. The findings support the notion that providers’ processes are mediated by individuals (patients, staff and healthcare professionals) with different capacities that influence their interactions [26]. For example, the evidence that providers’ organisational culture, geographic locations, and capacities related to patients’ income, cultural understandings of health and living situations (e.g. homelessness) can impact the provision or receipt of continuity.

## Implications for patients

Relational continuity is of paramount importance to patients’ experiences of continuity. The more frequently patients visit the GP, the more they value their relationship with them [33, 77]. This reflects a view of relational continuity as important in preventing and repairing gaps in care when transitioning across healthcare settings [40] as those with complex or chronic conditions do. It can also help them develop their credibility and candidacy for care for their issues to be taken seriously [90]. However, introducing named accountable GPs for patients alone may not translate into better continuity of care, assessed by the proportion of patient contacts with the named GP [87].

While informational continuity is perceived as an important pillar of patient care by healthcare professionals, this review suggests that informational continuity is also important to patients, particularly those with chronic conditions. Similarly, while management continuity was rarely discussed in the literature we reviewed, it was highlighted as important to patients with chronic conditions [69, 70], and increased need for care increases the risk of patients experiencing inadequate management continuity [9].

Patients’ expectations, needs and preferences often do not directly influence access or continuity of care. However, they may influence their likelihood of actively seeking continuity or accessing care if, for example, they do not trust their GP [79], particularly if they are unable to prioritise continuity due to the challenges of achieving (physical or timely) access [68, 84]. They are also highly pertinent to patients’ experiences of primary care and satisfaction with it. These factors are summarised in Table 3.

### Implications for providers and health system policy

With increasing patient demand and primary care workforce shortages in healthcare systems worldwide, access to a named GP may not be always achievable. However, a team of healthcare professionals taking responsibility for an allocated list of patients could improve both access and continuity [42, 60, 65]. In this context improving patients’ experiences of access and continuity of care requires engagement from a wider set of staff beyond GPs [65], including reception staff, who can facilitate appointment bookings for patients with their preferred GP [28]. There is a need to better understand what roles administrative and wider clinical staff, such as clinical pharmacists and physician associates, can play in maintaining access and continuity of care for patients [91].

This review also highlights that giving patients the option of telephone consultations [50], and longer hours during which healthcare professionals can be consulted by telephone [69] could both improve access and support continuity of care. A growing body of evidence supports the use of remote (email, video and telephone) consultations in general practice since the Covid-19 pandemic [92]. However, there is a need to evaluate the impact of remote consultations on the interpersonal relationships between patients and healthcare professionals and on relational continuity [92, 93]. While the increased use of remote appointment booking and consultation mechanisms removes a barrier to physical access for patients who face mobility or transport issues, it introduces barriers for those who are digitally excluded or those that rely on in-person interactions to support navigation of communication challenges, and could therefore undermine continuity of care. There is a need, therefore, to identify ways to best use remote consultations without widening health inequalities [94].

It is important to address issues of access and continuity because the lack of either has implications for the whole healthcare system. Patients who experience significant appointment delays may choose to opt out of consultations with their GP, and instead may seek care elsewhere, such as at an emergency department [36, 78]. In this regard, physical access can be viewed as a pre-requisite to other dimensions of access and to the possibility of relational continuity. It is a necessary condition without which the other elements cannot exist. Physical access is influenced by factors related to patients’ ability to pay for care within insurance-based health systems, and therefore to demographic factors related to deprivation, including ethnicity [57] and education level [34]. Where patient characteristics and circumstances, such as ability to navigate care, mediate access physical access, timely access or choice, they may also have a subsequent impact on relational continuity. It is dependent on the unique and evolving constellation of health system, practice and patient characteristics for any given patient. Therefore, relational continuity may fluctuate over time as the relationship between these mediating factors changes.

Our review demonstrates that physical access, timely access, and choice are moderated by health system, practice and patient characteristics. For any patient, provider access, and therefore relational continuity, may fluctuate over time as the constellation of mediating factors changes. Some of these characteristics are more enduring, such as the health system requiring patients to register, and the patient’s gender and ethnicity. Whereas others are dynamic and change with situational factors, such as where a patient lives in relation to their provider, their age and health conditions. This means that the notion of access and continuity as ‘fit’ [26] positions them as requiring patients to be treated as individuals, aligning with our call for a dynamic approach to continuity.

Finally, the literature highlights that patients want *both* timely access and choice [54, 74, 58, 95]. This means, first, providing physical access. Second, where timely access and choice come into conflict in relation to the urgency or acuteness of care needs then patients must be offered the option to prioritise quicker access on a given occasion or see a preferred healthcare professional. Finally, providers and professionals need to focus on providing access that fits patients’ individual needs in order to enable continuity of care, supported by practice policies that promote relational, management and informational continuity.

### Study strengths and limitations

To the authors’ knowledge this is the first systematic review that has focused on both access and continuity of care and how they are related. The paper addresses a gap by theorising this relationship, building on existing conceptual frameworks for access and continuity of care.

We did not conduct formal quality assessment of studies in this review as the articles because the literature was too heterogeneous to enable consistent and comparable quality appraisal. We minimised bias through involving two researchers in study inclusion decisions, data extraction and synthesis. The data and synthesis were discussed with the research team. Finally, findings from this study need to be interpreted in the context of differing national healthcare systems. The papers included in this scoping review had their own shortcomings. In particular, the preponderance of questionnaire studies inherently constrain understanding of the phenomena of access and continuity, and combined with the shortage of analysis of *both* patient and clinical perspectives, highlights the limits of our review on this basis. Nonetheless, the breadth of this scoping review allows us to derive insights about their relationship.

## Conclusions

This paper set out first to review the research literature exploring both access and continuity of care in primary care and, secondly, through synthesising this literature to determine what this means in terms of the relationship between access to and continuity of care. The literature review finds a range of factors at health system, provider and patient levels that influence provision of access and continuity of care and how they are experienced by patients. We have considered what our findings mean for patients and for primary care providers and health systems policy, and have outlined a model theorising the relationships between the dimensions of access and those of continuity. We conclude that far from two competing attributes of care, continuity and access to care are inseparable. This means that policies are needed that promote better access *in addition to* continuity of care rather than at its expense.

Further research is required to understand the inter-relationships between continuity and access under different circumstances and from the viewpoints of different stakeholders. Given the limitations of the studies reviewed in this paper, this research could profitably include observations of the work of reception staff booking patient appointments in addition to interviewing patients and clinicians to generate more nuanced understanding. The literature on continuity of care in primary care focuses on the GP-patient relationship and does not account for relational continuity arising from patients’ relationships with other healthcare professionals, such as nurses. Moreover, it rarely considers the other dimensions of continuity in Haggerty’s model (informational and management). Nonetheless, through examination of existing evidence we have developed a more nuanced model of the relationship between access and continuity in primary care which goes beyond existing rhetoric that these elements of patient experience are diametrically opposed. This can be used by policymakers, practitioners and researchers to help to identify ways to evaluate and improve both access and continuity of care for patients in primary care.

## Electronic supplementary material

Below is the link to the electronic supplementary material.

Supplementary Material 1

Supplementary Material 2

## Data Availability

No datasets were generated or analysed during the current study.

## Abbreviations

UK: United Kingdom
GP: General practitioner, family doctor or family physician
US: United States
NHS: English National Health Service

## References

[1] Levene LS, Baker RH, Newby C, Couchman EM, Freeman GK. Ongoing decline in continuity with gps in english general practices: A longitudinal study across the COVID-19 pandemic. Annals of Family Medicine. 2024;22(4):301–8. 10.1370/afm.3128 PMC11268676 38914438

[2] Jeffers H, Baker M. Continuity of care: still important in modern-day general practice. British Journal of General Practice. 2016;66(649):396–7. 27481958 10.3399/bjgp16X686185 PMC4979920

[3] Sheaff R, Halliday J, Øvretveit J, Byng R, Exworthy M, Peckham S, Asthana, S. Integration and continuity of primary care: polyclinics and alternatives – a patient-centred analysis of how organisation constrains care co-ordination. NIHR; 2015. 26312365

[4] Madan A, Manek N, Gregory S. General practice: the heart of the NHS. British Journal of General Practice. 2017;67(657):150. 28360043 10.3399/bjgp17X689965 PMC5565817

[5] Haggerty JL, Reid RJ, Freeman GK, Starfield BH, Adair CE, McKendry R. Continuity of care: a multidisciplinary review. BMJ (Clinical Res ed). 2003;327(7425):1219–21. 10.1136/bmj.327.7425.1219 PMC274066 14630762

[6] World Health Organization. Continuity and coordination of care: A practice brief to support implementation of the WHO framework on integrated people-centred health services. World Health Organization; 2018.

[7] van Walraven C, Oake N, Jennings A, Forster AJ. The association between continuity of care and outcomes: a systematic and critical review. Journal of Evaluation in Clinical Practice. 2010;16(5):947–56. 20553366 10.1111/j.1365-2753.2009.01235.x

[8] Pereira Gray DJ, Sidaway-Lee K, White E, Thorne A, Evans PH. Continuity of care with doctors—a matter of life and death? A systematic review of continuity of care and mortality. BMJ Open. 2018;8(6):e021161. 29959146 10.1136/bmjopen-2017-021161 PMC6042583

[9] Gulliford M, Cowie L, Morgan M. Relational and management continuity survey in patients with multiple long-term conditions. Journal of Health Services Research and Policy. 2011;16(2):67–74. 20592048 10.1258/jhsrp.2010.010015

[10] Baker R, Freeman GK, Haggerty JL, Bankart MJ, Nockels KH. Primary medical care continuity and patient mortality: a systematic review. British Journal of General Practice. 2020;70(698):e600–11. 32784220 10.3399/bjgp20X712289 PMC7425204

[11] Baker R, Streatfield J. What type of general practice do patients prefer? Exploration of practice characteristics influencing patient satisfaction. British Journal of General Practice. 1995;45(401):654–9. 8745863 PMC1239467

[12] House of Commons Health and Social Care Committee. The future of general practice. House of Commons; 2022. https://committees.parliament.uk.

[13] Benett IJ. Access, continuity, or both. British Journal of General Practice. 2014;64(625):388. 25071035 10.3399/bjgp14X680845 PMC4111315

[14] Boyle S, Appleby J, Harrison A. A rapid view of access to care. The King’s Fund; 2011.

[15] Goodall S, Montgomery A, Banks J, Salisbury C, Sampson F, Pickin M. Implementation of advanced access in general practice: postal survey of practices. British Journal of General Practice. 2006;56(533):918–23. 17132379 PMC1934051

[16] Salisbury C, Montgomery AA, Simons L, Sampson F, Edwards S, Baxter H, Goodall, S. Impact of advanced access on access, workload, and continuity: controlled before-and-after and simulated-patient study. British Journal General Practice. 2007;57(541):608–14. 17688754 PMC2099665

[17] Rosen R, Tomlinson J. How to improve continuity in general practice? [Internet]: Nuffield Trust. 2019. Available from: https://www.nuffieldtrust.org.uk/news-item/how-to-improve-continuity-in-general-practice

[18] Cook LL, Golonka RP, Cook CM, Walker RL, Faris P, Spenceley S, Lewanczuk, R, Wedel, R, Love, R, Andres, C, Byers, SD, Collins, T, Oddie, S. Association between continuity and access in primary care: a retrospective cohort study. CMAJ Open. 2020;8(4):E722–30. 33199505 10.9778/cmajo.20200014 PMC7676991

[19] Sayers LD. Access and continuity of care – the holy Grail of general practice. Rapid response to: Helen Salisbury: the complexity and joy of general practice. BMJ. 2022;376(0441). 10.1136/bmj.o441 35193892

[20] Uijen AA, Schers HJ, Schellevis FG, van den Bosch WJHM. How unique is continuity of care? A review of continuity and related concepts. Family Practice. 2012;29(3):264–71. 22045931 10.1093/fampra/cmr104

[21] Harper S. Continuity of care. AJN American Journal of Nursing. 1958;58(6):871–3. 13520811

[22] Shortell SM. Continuity of medical care: conceptualization and measurement. Medical Care. 1976;14(5):377–91. 1271879 10.1097/00005650-197605000-00001

[23] Levesque J-F, Harris MF, Russell G. Patient-centred access to health care: conceptualising access at the interface of health systems and populations. International Journal for Equity in Health. 2013;12(1):18. 23496984 10.1186/1475-9276-12-18 PMC3610159

[24] Simpson JM, Checkland K, Snow S, Voorhees J, Rothwell K, Esmail A. Access to general practice in England: time for a policy rethink. British Journalof General Practice. 2015;65(640):606–7. 26500315 10.3399/bjgp15X687601 PMC4617262

[25] Andersen RM, McCutcheon A, Aday LA, Chiu GY, Bell R. Exploring dimensions of access to medical care. Health Service Research. 1983;18(1):49–74. 6841113 PMC1068709

[26] Voorhees J, Bailey S, Waterman H, Checkland K. Advancing an Understanding of access as ‘human fit’: A qualitative participatory case study in general practice. British Journal of General Practice. 2022 10.3399/BJGP.2021.0375 PMC8843400 34990392

[27] NHS. GP Patient survey. Available from: https://www.gp-patient.co.uk/

[28] Alazri M, Heywood P, Leese B. How do receptionists view continuity of care and access in general practice? European Journal of General Practice. 2007;13(2):75–82. 17534743 10.1080/13814780701379048

[29] Oliver D, Deal K, Howard M, Qian H, Agarwal G, Guenter D. Patient trade-offs between continuity and access in primary care interprofessional teaching clinics in Canada: A cross-sectional survey using discrete choice experiment. BMJ Open. 2019;9(3). 10.1136/bmjopen-2018-023578 PMC6475162 30904840

[30] Gill N, Freeman GK. Continuity of care and rapid access: the potential impact of appointment systems. Primary Health Care Research and Development. 2007;8(3):235–42.

[31] Mays N, Pope C, Popay J. Systematically reviewing qualitative and quantitative evidence to inform management and policy-making in the health field. J Health Services Research and Policy. 2005;10(1suppl):6–20. 16053580 10.1258/1355819054308576

[32] QSR International Pty Ltd. NVivo (Version 12). QSR International Pty Ltd; Available from: https://www.qsrinternational.com/nvivo-qualitative-data-analysis-software/home

[33] Kearley KE, Freeman GK, Heath A. An exploration of the value of the personal doctor-patient relationship in general practice. British Journal of General Practice. 2001;51(470):712–8. 11593831 PMC1314098

[34] Stoddart H, Evans M, Peters TJ, Salisbury C. The provision of ‘same-day’ care in general practice: an observational study. Family Practice. 2003;20(1):41–7. 12509369 10.1093/fampra/20.1.41

[35] Boulton M, Tarrant C, Windridge K, Baker R, Freeman GK. How are different types of continuity achieved? A mixed methods longitudinal study. British Journal of General Practice. 2006;56(531):749–55. 17007704 PMC1920714

[36] Guthrie B, Wyke S. Personal continuity and access in UK general practice: A qualitative study of general practitioners’ and patients’ perceptions of when and how they matter. BMC Family Practice. 2006;7. 10.1186/1471-2296-7-11 PMC1413534 16504130

[37] Turner D, Tarrant C, Windridge K, Bryan S, Boulton M, Freeman G, Baker, R. Do patients value continuity of care in general practice? An investigation using stated preference discrete choice experiments. Journal of Health Services Research and Policy. 2007;12(3):132–7. 17716414 10.1258/135581907781543021

[38] Cheraghi-Sohi S, Hole AR, Mead N, McDonald R, Whalley D, Bower P, Roland, M. What patients want from primary care consultations: A discrete choice experiment to identify patients’ priorities. Annals of Family Medicine. 2008;6(2):107–15. 18332402 10.1370/afm.816 PMC2267425

[39] Paddison CAM, Saunders CL, Abel GA, Payne RA, Adler AI, Graffy JP, Roland, MO. How do people with diabetes describe their experiences in primary care? Evidence from 85,760 patients with self-reported diabetes from the english general practice patient survey. Diabetes Care. 2015;38(3):469–75. 25271208 10.2337/dc14-1095

[40] Tarrant C, Windridge K, Baker R, Freeman G, Boulton M. Falling through gaps’: primary care patients’ accounts of breakdowns in experienced continuity of care. Family Practice. 2015;32(1):82–7. 25411422 10.1093/fampra/cmu077 PMC5926435

[41] Forbes LJL, Forbes H, Sutton M, Checkland K, Peckham S. Changes in patient experience associated with growth and collaboration in general practice: observational study using data from the UK GP patient survey. British Journal of General Practice. 2020;70(701):e906. 33139333 10.3399/bjgp20X713429 PMC7643819

[42] Murphy M, Salisbury C. Relational continuity and patients’ perception of GP trust and respect: a qualitative study. British Journal of General Practice. 2020;70(698):e676–83. 32784221 10.3399/bjgp20X712349 PMC7425201

[43] Bower P, Roland M, Campbell J, Mead N. Setting standards based on patients’ views on access and continuity: secondary analysis of data from the general practice assessment survey. BMJ. 2003;326(7383):258–60. 12560279 10.1136/bmj.326.7383.258 PMC140766

[44] Lester H, Tritter JQ, Sorohan H. Patients’ and health professionals’ views on primary care for people with serious mental illness: focus group study. BMJ. 2005;330(7500):1122. 10.1136/bmj.38440.418426.8F PMC557894 15843427

[45] Slater J, Malik S, Davey P, Grant S. Improving access to primary care: A mixed-methods approach studying a new review appointment system in a Scottish GP practice. BMJ Open Quality. 2021;10(2). 10.1136/bmjoq-2020-001279 PMC8237728 34172510

[46] Voorhees J, Bailey S, Waterman H, Checkland K. Accessing primary care and the importance of ‘human fit’: a qualitative participatory case study. British Journal of General Practice. 2022;72(718):E342–50. 34990392 10.3399/BJGP.2021.0375 PMC8843400

[47] Nair KM, Dolovich LR, Ciliska DK, Lee HN. The perception of continuity of care from the perspective of patients with diabetes. Family Medicine. 2005;37(2):118–24. 15690252

[48] Haggerty JL, Pineault R, Beaulieu MD, Brunelle Y, Gauthier J, Goulet F, Rodrigue, J. Room for improvement: patients’ experiences of primary care in Quebec before major reforms. Canadian Family Physician. 2007;53(6):1057, 2001:e.1–6, 1056. PMC1949223 17872786

[49] Haggerty J, Burge F, Levesque J-F, Gass D, Pineault R, Beaulieu M-D, Santor, D. Operational definitions of attributes of primary health care: consensus among Canadian experts. Annals of Family Medicine. 2007;5(4):336–44. 17664500 10.1370/afm.682 PMC1934980

[50] Haggerty JL, Pineault R, Beaulieu MD, Brunelle Y, Gauthier J, Goulet F, Rodrigue, J.. Practice features associated with patient-reported accessibility, continuity, and coordination of primary health care. Annals of Family Medicine. 2008;6(2):116–23. 18332403 10.1370/afm.802 PMC2267415

[51] Wetmore S, Boisvert L, Graham E, Hall S, Hartley T, Wright L, Hammond, J, Ings, H, Pawelec-Brzychczy, A, Valiquet. Patient satisfaction with access and continuity of care in a multidisciplinary academic family medicine clinic. Canadian Family Physician. 2014;60(4):e230–6. 24733343 PMC4046539

[52] Pineault R, Provost S, Borgès Da Silva R, Breton M, Levesque JF. Why is bigger not always better in primary health care practices?? The role of mediating organizational factors. Inquiry. 2016;53. 10.1177/0046958015626842 PMC5798712 26831624

[53] Lamanna D, Stergiopoulos V, Durbin J, O’Campo P, Poremski D, Tepper J. Promoting continuity of care for homeless adults with unmet health needs: the role of brief interventions. Health and Social Care in the Community. 2018;26(1):56–64. 10.1111/hsc.12461 28569397

[54] Marshall EG, Wuite S, Lawson B, Andrew MK, Edwards L, MacKenzie A, Woodrow, AC, Peddle, S. What do you mean I can’t have a Doctor?? This is Canada! – a qualitative study of the myriad consequences for unattached patients awaiting primary care attachment. BMC Primary Care. 2022;23(1). 10.1186/s12875-022-01671-5 PMC8966849 35354438

[55] Smithman MA, Haggerty J, Gaboury I, Breton M. Improved access to and continuity of primary care after attachment to a family physician: longitudinal cohort study on centralized waiting lists for unattached patients in Quebec, Canada. BMC Primary Care. 2022;23(1). 10.1186/s12875-022-01850-4 PMC9482231 36114464

[56] Del Grande C, Kaczorowski J, Pomey MP. What are the top priorities of patients and clinicians for the organization of primary cardiovascular care in Quebec? A modified e-Delphi study. PLoS ONE. 2023;18(1 January). 10.1371/journal.pone.0280051 PMC9812320 36598919

[57] Forrest CB, Starfield B. Entry into primary care and continuity: the effects of access. American Journal of Public Health. 1998;88(9):1330–6. 9736872 10.2105/ajph.88.9.1330 PMC1509069

[58] Forrest CB, Shi LY, von Schrader S, Ng J. Managed care, primary care, and the patient-practitioner relationship. Journal of General Internal Medicine. 2002;17(4):270–7. 11972723 10.1046/j.1525-1497.2002.10309.x PMC1495029

[59] Locatelli SM, Hill JN, Talbot ME, Schectman G, LaVela SL. Relational continuity or rapid accessibility in primary care? A mixed-methods study of veteran preferences. Quality Management in Health Care. 2014;23(2):76–85. 24710183 10.1097/QMH.0000000000000028

[60] Panattoni L, Stone A, Chung S, Tai-Seale M. Patients report better satisfaction with Part-Time primary care physicians, despite less continuity of care and access. Journal of General Internal Medicine. 2015;30(3):327–33. 25416600 10.1007/s11606-014-3104-6 PMC4351271

[61] Rosland AM, Krein SL, Kim HM, Greenstone CL, Tremblay A, Ratz D, Saffar, D. Measuring patient-centered medical home access and continuity in clinics with part-time clinicians. American Journal of Managed Care. 2015;21(5):e320–8. 26167780

[62] Hung D, Chung S, Martinez M, Tai-Seale M. Effect of organizational culture on patient access, care continuity, and experience of primary care. Journal of Ambulatory Care Management. 2016;39(3):242–52. 27232685 10.1097/JAC.0000000000000116

[63] Weir SS, Page C, Newton WP. Continuity and access in an academic family medicine center. Family Medicine. 2016;48(2):100–7. 26950780

[64] Ehman KM, Deyo-Svendsen M, Merten Z, Kramlinger AM, Garrison GM. How preferences for continuity and access differ between Multimorbidity and healthy patients in a team care setting. Journal of Primary Care Community Health. 2017;8(4):319–23. 28434390 10.1177/2150131917704556 PMC5932726

[65] Forman JH, Robinson CH, Krein SL. Striving toward team-based continuity: provision of same-day access and continuity in academic primary care clinics. BMC Health Services Research. 2019;19(1). 10.1186/s12913-019-3943-2 PMC6399842 30832649

[66] Norwood P, Correia I, Heidenreich S, Veiga P, Watson V. Is relational continuity of care as important to people as policy makers think? Preferences for continuity of care in primary care. Family Practice. 2021. 10.1093/fampra/cmab010 33738479

[67] Norwood P, Correia I, Veiga P, Watson V. Patients’ experiences and preferences for primary care delivery: a focus group analysis. Primary Health Care Research and Development. 2019;20:e106. 32799999 10.1017/S1463423619000422 PMC8060815

[68] Krzton-Krolewiecka A, Oleszczyk M, Windak A. Do Polish primary care physicians Meet the expectations of their patients? An analysis of Polish QUALICOPC data. BMC Family Practice. 2020;21(1):118. 32576153 10.1186/s12875-020-01190-1 PMC7313208

[69] Kuipers SJ, Nieboer AP, Cramm JM. Making care more patient centered; experiences of healthcare professionals and patients with Multimorbidity in the primary care setting. BMC Family Practice. 2021;22(1):1–15. 33836652 10.1186/s12875-021-01420-0 PMC8035730

[70] Kuipers SJ, Nieboer AP, Cramm JM. Views of patients with multi-morbidity on what is important for patient-centered care in the primary care setting. BMC Family Practice. 2020;21(1). 10.1186/s12875-020-01144-7 PMC7184691 32336277

[71] Homburg M, Brandenbarg D, Hartman TO, Ramerman L, Beugel G, Rijpkema C, Verheij, R, Berger, M, Peters, L. Patient experiences during the COVID-19 pandemic: a qualitative study in Dutch primary care. BJGP Open. 2022;6(4). 10.3399/BJGPO.2022.0038 PMC9904784 36270671

[72] Hjortdahl P. General practice and continuity of care: organizational aspects. Family Practice. 1989;6(4):292–8. 2632307 10.1093/fampra/6.4.292

[73] von Bültzingslöwen I, Eliasson G, Sarvimäki A, Mattsson B, Hjortdahl P. Patients’ views on interpersonal continuity in primary care: A sense of security based on four core foundations. Family Practice. 2006;23(2):210–9. 16361395 10.1093/fampra/cmi103

[74] Droz M, Senn N, Cohidon C. Communication, continuity and coordination of care are the most important patients’ values for family medicine in a fee-for-services health system. BMC Family Practice. 2019;20(1). 10.1186/s12875-018-0895-2 PMC6346577 30683051

[75] Altin SV, Stock S. Impact of health literacy, accessibility and coordination of care on patient’s satisfaction with primary care in Germany. BMC Family Practice. 2015;16(1). 10.1186/s12875-015-0372-0 PMC4619202 26492959

[76] Lautamatti E, Mattila K, Suominen S, Sillanmäki L, Sumanen M. A named GP increases self-reported access to health care services. BMC Health Services Research. 2022;22(1). 10.1186/s12913-022-08660-5 PMC9580200 36261827

[77] Kenny P, De Abreu Lourenco R, Wong CY, Haas M, Goodall S. Community preferences in general practice: important factors for choosing a general practitioner. Health Expectations: International Journalof Public Participation inHealth Care and Health Policy. 2016;19(1):26–38. 10.1111/hex.12326 PMC5055222 25565251

[78] Sav A, McMillan SS, Kelly F, King MA, Whitty JA, Kendall E, Wheeler, AJ. The ideal healthcare: priorities of people with chronic conditions and their carers. BMC Health Services Research. 2015;15(1). 10.1186/s12913-015-1215-3 PMC4678633 26666351

[79] Reid J, Cormack D, Crowe M. The significance of relational continuity of care for Māori patient engagement with predominantly non-Māori Doctors: findings from a qualitative study. Austrialian and New Zealand Journal of Public Health. 2016;40(2):120–5. 26337777 10.1111/1753-6405.12447

[80] Jego M, Grassineau D, Balique H, Loundou A, Sambuc R, Daguzan A, Gentile, G, Gentile, S. Improving access and continuity of care for homeless people: how could general practitioners effectively contribute? Results from a mixed study. BMJ Open 2016;6(11). 10.1136/bmjopen-2016-013610 PMC5168510 27903566

[81] De Foo C, Surendran S, Jimenez G, Ansah JP, Matchar DB, Koh GCH. Primary care networks and Starfield’s 4cs: A case for enhanced chronic disease management. Int Journal of Environmental Research and Public Health. 2021;18(6):1–14. 10.3390/ijerph18062926 PMC8001119 33809295

[82] Antoun JM, Hamadeh GN, Adib SM. What matters in the patients’ decision to revisit the same primary care physician? Lebanese Medical Journal. 2014;62(4):198–202. 25807716 10.12816/0008287

[83] Abreu DMX, Araújo LHL, Reis C, Lima ÂMLD, Santos AFD, Jorge AO, Sobrinho, DF, Gonzaga da Matta Machado, AT. Service users’ perception about healthcare provided by teams participating in the National program for primary care access and quality improvement in Brazil. Epidemiological Services Saude. 2018;27(3):e2017111. 30183866 10.5123/S1679-49742018000300002

[84] Wang W, Zhao R, Zhang J, Xu T, Lu J, Nicholas S, Wei, X, Lui, X, Yang, H, Maitland, E. Public expectations of good primary health care in China: a national qualitative study. Family Practice. 2022. 10.1093/fampra/cmac149 36573339

[85] Kringos D, Boerma W, Bourgueil Y, Cartier T, Dedeu T, Hasvold T, Hutchinson, A, Lember, M, Oleszczyk, M, Pavlic, RP, Svab, I, Tedeschi, P, Wilm, S, Wilson, A, Windak, A, Van der Zee, J, Groenewegen, P. The strength of primary care in Europe: an international comparative study. British Journal General Practice. 2013;63(616):e742 24267857 10.3399/bjgp13X674422 PMC3809427

[86] Gray DP, Sidaway-Lee K, Evans P. Continuity of GP care: using personal lists in general practice. British Journal General Practice. 2022;72(718):208. 35483941 10.3399/bjgp22X719237 PMC11189035

[87] Barker I, Lloyd T, Steventon A. Effect of a National requirement to introduce named accountable general practitioners for patients aged 75 or older in England: regression discontinuity analysis of general practice utilisation and continuity of care. BMJ Open. 2016;6(9):e011422. 27638492 10.1136/bmjopen-2016-011422 PMC5030554

[88] Pandhi N, Saultz JW. Patients’ perceptions of interpersonal continuity of care. Journal American Board of Family Medicine. 2006;19(4):390–7. 10.3122/jabfm.19.4.390 16809654

[89] Gardner K, Banfield M, McRae I, Gillespie J, Yen L. Improving coordination through information continuity: a framework for translational research. BMC Health Services Research. 2014;14(1):590. 25421916 10.1186/s12913-014-0590-5 PMC4245827

[90] Rhodes P, Sanders C, Campbell S. Relationship continuity: when and why do primary care patients think it is safer? British Journal of General Practice. 2014;64(629):e758–64. 25452540 10.3399/bjgp14X682825 PMC4240148

[91] Wiltshire A. 2019. Available from: https://www.health.org.uk/news-and-comment/blogs/improving-continuity-of-care-in-general-practice-four-lessons-from-the

[92] Mold F, Cooke D, Ip A, Roy P, Denton S, Armes J. COVID-19 and beyond: virtual consultations in primary care—reflecting on the evidence base for implementation and ensuring reach:commentary article. BMJ Health & Care Informatics. 2021;28(1):e100256. 10.1136/bmjhci-2020-100256 PMC7804830 33436372

[93] Randhawa RS, Chandan JS, Thomas T, Singh S. An exploration of the attitudes and views of general practitioners on the use of video consultations in a primary healthcare setting: a qualitative pilot study. Primary Health Care Research Development. 2019;20:e5. 29909798 10.1017/S1463423618000361 PMC6476389

[94] Mold F, Hendy J, Lai Y-L, de Lusignan S. Electronic consultation in primary care between providers and patients: systematic review. JMIR Medicine Informatcs. 2019;7(4):e13042. 10.2196/13042 PMC6918214 31793888

[95] Gerard K, Salisbury C, Street D, Pope C, Baxter H. Is fast access to general practice all that should matter? A discrete choice experiment of patients’ preferences. J Health Services Research and Policy. 2008;13(SUPPL 2):3–10. 18416923 10.1258/jhsrp.2007.007087

