# Supplementary material for: Access or continuity: a zero sum game? A systematic review of the literature examining the relationship between access and continuity in primary healthcare
Source: BMC Prim Care. 2025 Jul 2;26:202. doi: 10.1186/s12875-025-02860-8 (PMC12217832; doi:10.1186/s12875-025-02860-8)
Supplement: Supplementary file 2 — Supplementary Material 2 [file 12875_2025_2860_MOESM2_ESM.docx]

**Appendix 1** Summary of included studies

| Author(s) & year | Country | Study aim(s) | | Study design | | Sample | | How was access/ continuity measured/conceived? | | Relevant findings | | Themes | | Relationship between access & continuity | |
| --- | --- | --- | --- | --- | --- | --- | --- | --- | --- | --- | --- | --- | --- | --- | --- |
| Hjortdahl, P. (1989) | Norway | To evaluate organizational factors influencing continuity of care in Norwegian general practice | | Questionnaire | | 207 general practitioners randomly drawn from list of physicians held by the Norwegian Medical Association in 1986 | | General practitioners' accessibility to their patients by telephone, during regular daytime working hours, when doing consultations.  General practitioners' accessibility to their patients during the working day.  General practitioners’ contact with patients during treatment. | | Doctors in single handed practices were significantly more available to patient requests than colleagues in practices with two or more principals, regardless of the kind of medical work.  Another factor influencing accessibility was the staffing of the office when the doctor was away. In these situations, 187 (90%) of the practices were staffed by another doctor, nurse or receptionist. Among the 47 single handed practices, 58% were staffed by a nurse or receptionist, 21% by a telephone answering machine and 21% did not have any specific arrangements for patients to get in touch. | | “System-level factors that influence access and continuity of care”; “practice-level factors that influence access and continuity of care”; “what is important for patients?” | | **Personal lists and practice staffing influenced physical access and relational continuity.** | |
| Baker et al. (1995) | UK | To examine the characteristics of general practices that influence patient satisfaction. | | Questionnaire | | 220 patients in 89 general practices. | | Access: ease of getting to the general practice, appointments with doctors, telephone service.  Continuity: seeing the same general practitioner. | | Increasing total list size of patients registered with practices was associated with decreasing levels of general satisfaction and decreased satisfaction with accessibility, availability and continuity of care.  The presence of a personal list system was associated with increased levels of general satisfaction and increased satisfaction with accessibility and continuity of care.  Training practices were associated with decreased levels of general satisfaction and decreased satisfaction with availability and continuity of care. | | “System-level factors that influence access and continuity of care”; “practice-level factors that influence access and continuity of care”; “what is important for patients?” | | **Increasing total list size of patients reduced relational continuity, physical and timely access. Whereas having a personal list increased both relational continuity, physical and timely access.** | |
| Forrest et al. (1998) | US | Examine the relationship between access and use of primary care physicians as sources of first contact and continuity with the medical system. | | Questionnaire | | 19,835 individuals who responded to the 1987 National  Medical Expenditure Survey | | Access:   - geographic ability to travel to the primary source of care - Financial costs associated with health care-seeking - organizational-factors related to arranging encounters between patients and physician.   Continuity: doctor the participant usually sees at the primary care site. | | The multivariate linear regression model demonstrated that individuals with access barriers in out of hours care, appointment wait, and lack of insurance had significantly lower levels of continuity of care. | | “System-level factors that influence access and continuity of care”; “what is important for patients?”. | | **Barriers to timely and physical access led to lower levels of relational continuity.** | |
| Kearley et al. (2001) | UK | To determine how many patients report having a personal doctor and when this is most valued  To compare the value of a personal doctor–patient relationship with that of convenience | | Patient and GP Questionnaire | | A total of 284 GPs and 996 patients from a stratified random sample of 18 Oxfordshire practices | | *Patient questionnaire*  Continuity: questions about having a personal GP and priority for seeing such a GP for 13 clinical scenarios.  Access: a four-point scale to rate how easy it was to see whichever GP they want to when they visit the practice’ that day, within three days or within one week.  *GP questionnaire*  Continuity: asked questions about the GP/patient relationship similar to those in the patient questionnaire. | | Overall, 75% of patients reported having at least one personal GP.  In the clinical cameos concerning more important problems, the overwhelming majority of patients valued having a personal GP more than a convenient appointment. A convenient appointment time was less important ‘in general’ to patients than having a personal GP.  Personal care was more highly valued than convenience by the great majority of patients and GPs for more important problems | | “What is important for patients?” | | **Patients preferred relational continuity over timely access for a number of clinical scenarios with the exception of cold, itchy rash, painful ear or painful knee.** | |
| Forrest et al. (2002) | US | Examine how specific attributes of managed health plans influence patients' relationships with their primary care practitioners (PCPs) and determine whether these effects are mediated by access to, continuity with, or perceived choice of PCPs. | | Questionnaire | | 19,415 patients whose most recent visit in the past 12 months was made to their primary care delivery site. | | Continuity: with the primary care site was examined using 2 items. The first divided individuals into those who usually see the same clinician at the primary care site versus those who do not. The second item separated the sample into patients whose relationship with their primary care source was <12 months and ≥12 months.  Access:   - travel time to the primary care site (geographic access) - length of time the individual waited for an appointment - length of office wait. | | Shorter office waits, having a specific clinician at the primary care site, better perceived choice of PCPs, and a longer duration of relationship with the primary care practitioner were associated with higher ratings of the patient-PCP relationship.  Poorer patient-PCP relationships among uninsured adults were mediated in part by less access to and continuity with primary care practitioners. | | “What is important for patients?”. | | **Timely access, choice of professional and relational continuity, were associated with better patient-provider relationship.** | |
| Stoddart et al. (2003) | UK | To investigate the provision of ‘same-day’ care and the characteristics, treatment expectations and priorities of patients attending for ‘routine’ and ‘same-day’ appointments. | | Questionnaire | | 362 people attending same-day, and 362 people attending routine appointments, at 15 general practices. | | The uptake of same-day and routine appointments was determined from appointment system records.  Treatment expectations and preferences were explored by asking patients to respond in three ways to two illness scenarios, one describing an acute illness (sore throat) and one a chronic illness (back pain). First, patients were asked what sources of help they would consider in response to each scenario. Secondly, they were asked the extent to which they agreed or disagreed with statements relating to seeing a doctor quickly, seeing a doctor they knew and seeing a doctor rather than a nurse, using Likert-type scales. Thirdly, they ranked these three issues in order of importance. | | In univariable analyses, use of same-day care was greater by people who were younger, non-white, in work and with educational qualifications. From multivariable analyses, only age, speed of previously obtaining appointments, expecting a prescription and having the problem for a short time were independently associated with receiving same-day care.  Rapid access to care was rated as more important than seeing a known doctor for two clinical scenarios. There was no difference between scenarios for seeing a doctor rather than a nurse. | | “What is important for patients?”. | | **Timely access was preferred over relational continuity in sore throat and back pain scenarios.** | |
| Bower et al. (2003) | UK | To examine relations between reports of access and continuity in general practice and assessments of acceptability to derive patient-based standards and to examine differences in standards between patients from different sociodemographic group. | | Secondary analysis | | Data from general practice research studies and routine quality assessment activities undertaken by practices and primary care trusts. | | Used access and continuity items on the general practice assessment survey.  Access: waiting times for appointments with a particular general practitioner, with any general practitioner, and for consultations to begin.  Continuity: the proportion of consultations with the patient's usual general practitioner (scale - Always; Almost always; A lot of the time; Some of the time; Almost never; Never). | | Satisfactory standards of access were next day appointments with general practitioners and a 6–10 minute wait for consultations to begin. A satisfactory level of continuity was seeing the same general practitioner “a lot of the time”. | | “What is important for patients?” | | **In relation to timely access next day appointments were considered satisfactory by patients. In terms of relational continuity, seeing the same GP “a lot of the time” was considered satisfactory.** | |
| Lester et al. (2005) | UK | To explore the experience of providing and receiving primary care from the dual perspectives of primary care health professionals and patients with serious mental illness. | | Focus groups | | 54 patients with serious mental illness, 39 general practitioners (GPs), and 8 practice nurses. | | See relevant findings | | Most patients preferred to consult their own GP, who listened and was willing to learn, rather than be referred to a different GP with specific mental health knowledge.  Swift access was important to patients, with barriers created by the effects of the illness and the noisy or crowded waiting area. Although general practitioners felt that lack of knowledge inhibits greater involvement in care, patients valued continuity of care, listening skills, and willingness to learn more than specific knowledge about mental health. | | “What is important for patients?”; “promoting access and continuity of care in general practice”. | | **Mental health patients preferred timely access and relational continuity when receiving primary care services.** | |
| Nair et al. (2005) | Canada | Explored the meaning of continuity of care for patients with diabetes and the factors that enhance or detract from continuity of care from the patients’ perspective. | | Focus groups | | Seven focus groups with 46 adult patients were held at a health service organization in Northern Ontario. | | See relevant findings | | Patients conceptualized continuity of care in a broad and multifaceted way as comprised of five components: (1) access to services, (2) interactions with physician, (3) interactions with other health care providers, (4) personal self-responsibility, and (5) communication.  Factors that enhance continuity of care: (1) The physician is able to provide health care services that are regular, timely, and efficient. (2) The patient can access specialists and have medical tests done when needed.  Factors that detract from continuity of care: (1) The patient experiences long wait times and cancelled appointments (2) The patient experiences a lack of available providers and appointments are not predictable. | | “What is important for patients?”; “promoting access and continuity of care in general practice”. | | **Timely access to GP enhanced relational continuity.**  **On the other hand, barriers to timely access (long waiting times) and physical access (availability of providers, nature of appointments) also hindered relational continuity.** | |
| Boulton et al. (2006) | UK | To explore how patients regard and use primary care services in relation to continuity of provider and access to care, to identify factors that promote or hinder their success in achieving their preferences, and to describe what this means for how different types of continuity are achieved. | | Mixed methods (in-depth interviews, consultation record booklets completed by patients over 6 months and general practice records for the year including the study period). | | Purposive sample of 31 patients recruited from general practices, walk-in centres and direct advertising | | See relevant findings | | Some participants had preference for and success in seeing a named provider for some problems; priority given to and success in obtaining swift access to care for others. These participants include young parents who made an effort to see their ‘own’ GP for their own problems but who looked for quick access to any appropriate provider for their children; and patients who made an effort to see their ‘own’ GP for chronic health problems but gave a greater priority to quick or convenient access when they experienced minor acute problems. | | “Promoting access and continuity of care in general practice”. | | **Preference for timely access over relational continuity and vice-versa was context/condition/case specific.** | |
| Guthrie et al (2006) | UK | Examined patient and general practitioner (GP) perceptions of the value of personal continuity and rapid access, and the relationship between them. | | Semi-structured interviews | | 6 GPs and 32 patients in the Lothian region of Scotland | | See relevant findings | | From the patients' perspective, what mattered was 'access to appropriate care' depending on the problem to be dealt with. For a few patients, rapid access was the only priority. For most, rapid access was balanced against greater involvement in the consultation when seeing 'their' trusted doctor, which was particularly valued for chronic, complex and emotional problems. GPs did not perceive enabling access to be a core part of their work. There was little evidence that GPs routinely discussed with patients when or how personal continuity and access should be balanced. | | “What is important for patients?”; “promoting access and continuity of care in general practice”. | | **Most patients wanted timely access balanced with effective relational continuity.**  **GPs and patient views on the importance of relational continuity and timely access were not aligned.** | |
| Von Bültzingslöwen et al. (2006) | Sweden | To acquire a comprehensive understanding of the core values of having a personal doctor in a continuing doctor–patient relationship in primary care among long-term, chronically ill patients. | | Semi-structured interviews | | 14 chronically ill patients & 16 health care professionals at three primary health care centres | | See relevant findings | | Both patients and staff emphasized the security that comes from knowledge about the doctor and his or her way of working and ‘way of being’. For patients to know what can be expected, reinforced an overall feeling of security.  It was important to patients to be able to reach the doctor. Personal GPs had noticed that their patients felt secure if they knew how to get in touch with their doctor. | | “What is important for patients?”; “promoting access and continuity of care in general practice”. | | **Knowing how to access a personal GP (physical access) increased the strength of relational continuity** | |
| Alazri et al. (2007) | UK | To explore how receptionists might influence access and continuity of care in general practice. | | Questionnaire | | 148 receptionists | | - Receptionists’ definitions of continuity - how practice policies influenced receptionists’ decisions when making appointments - receptionists’ responses when offering appointments to patients in urgent and non-urgent circumstances - receptionists’ responses when offering appointments to patients with special circumstances (chronic conditions, the elderly, sick children) - how receptionists responded when patients did not attend their appointments. | | The majority of receptionists perceived continuity as team continuity. Most felt it was important for the patient to be seen on the same day by any doctor, rather than the usual doctor. They were less willing to ask patients for more details of a routine problem than an urgent one. Organizational factors affected how receptionists offered appointments. | | “System-level factors that influence access and continuity of care” | | **How receptionists offered appointments to patients impacted timely access and relational continuity** | |
| Gill et al. (2007) | UK | To investigate how limiting the option of advance booking affected patients’ scope to choose whom they see. | | Questionnaire | | 651 patients at three of the largest group practices in a West London Primary Care Trust | | Access:  Practices A and B encouraged patients to book appointments by telephone. All patients who wanted to be seen sooner than the first available appointment were telephoned by a ‘triage’ doctor who would negotiate advice, a prescription, a later appointment, or a same-day consultation – usually with themselves.  Practice C operated what they described as an ‘advanced access’ appointment system. A limited number of appointments were bookable up to one week ahead. All other consulting times were reserved for same day booking.  Continuity was assessed in a questionnaire with items covering whether the patient saw the doctor of their choice; their choice of doctor; whether they had a usual doctor; how well their usual doctor knew their medical history; how much they trusted their usual doctor. | | While most patients in the so- called ‘advanced access’ practice were seen very quickly, fewer patients, especially those with chronic conditions, were able to see their chosen doctor than in two practices offering more scope for booking appointments ahead. | | “System-level factors that influence access and continuity of care” | | **Prioritising same day access over advance booking improved timely access at the expense of relational continuity.** | |
| Haggerty, Pineault et al. (2007) | Canada | To investigate variations in accessibility, continuity of care, and coordination of services as experienced by patients in Quebec on the eve of major reforms, and to provide baseline information against which reforms could be measured. | | Questionnaire | | 3441 patients from 100 primary health care clinics in Quebec, Canada | | Patients’ experiences were assessed principally through the Primary Care Assessment Tool (PCAT), which measures strength of patient-provider affiliation, degree of confidence in being seen within a day for a new problem (first-contact accessibility), confidence that providers know the medical history and personal situation and will manage care on an ongoing basis (relational continuity) and work well with specialists (coordination of care), and patients’ recall of having received preventive care. | | Most participants reported they were highly confident that their physicians knew them well and would manage their care beyond clinical encounters.  Only 10.4% pf participants expressed confidence they could be seen within a day by their regular providers.  While 85% rated convenience of office location as very good or excellent, only 48% rated waiting time for an appointment as very good or excellent, and only 46% rated ease of contacting a physician by telephone as very good or excellent.  Relational continuity, accumulated knowledge, and care management were well within acceptable levels.  Not surprisingly, patients without regular physicians had significantly less confidence in these aspects of care than those with regular physicians did | | “System-level factors that influence access and continuity of care” | | **Patients without regular physicians had less confidence in receiving timely access; physical access; relational continuity.** | |
| Haggerty, Burge et al. (2007) | Canada | To define the attributes that should be evaluated in predominant and proposed models of primary health care in the Canadian context. | | Delphi study | | Twenty persons considered to be experts in primary health care | | See relevant findings | | This consensus process resulted in 25 operational definitions of attributes of primary health care. Only a few were identified as being specific to primary health care: first-contact accessibility, relational continuity, family-centred care, population orientation, and intersectoral teamwork. | | “Promoting access and continuity of care in general practice”. | | **Timely access and relational continuity were considered to be important attributes of primary care.** | |
| Salisbury et al. (2007) | UK | To determine whether implementation of Advanced Access in general practice is associated with benefits | | Controlled before-and-after and simulated-patient study. In a ‘simulated patient’ study, the researchers telephoned each practice once a month for 11 consecutive months to make an appointment with a doctor. | | Twenty-four practices that had implemented Advanced Access and 24 that had not. | | Access:   - Each attempt to contact the practice involved: a request for an appointment with any doctor and five with a randomly selected named doctor. - The time taken to make telephone contact - The wait for the first and the third available appointments were recorded. If the receptionist was not able to offer any appointments, offered alternatives were recorded.   Continuity: analysed using the Continuity of Care index. | | Practices operating Advanced Access provided slightly quicker access to an appointment than control practices, with no evidence that there was any difference in total capacity, workload, or continuity of care. | | “System-level factors that influence access and continuity of care” | | **Prioritising same day access over advance booking ahead increased timely access without hindering relational continuity.** | |
| Turner et al. (2007) | UK | To estimate the relative importance to patients of continuity of care compared with other aspects of a primary care consultation. | | Discrete choice experiment & interviews | | Stratified random sample of 646 community dwelling adults taken from general practitioner (GP) registers, plus 20 interviews with Punjabi, Urdu and Gujarati speakers. | | Access: length of time in days patients have to wait for a consultation.  Continuity: Seeing a GP; seeing a person you know and trust; seeing a person who has information about your medical history. | | Respondents stated their preference to wait longer to see a familiar medical practitioner who was well informed about their case when they had a problem causing uncertainty or needed a routine check- up. They preferred quick access for likely minor ‘low impact’ symptoms. | | “What is important for patients?” | | **Preference for timely access over relational continuity and vice-versa was context/condition specific.** | |
| Cheraghi-Sohi et al. (2008) | UK | To assess patients’ priorities for a range of attributes of primary care consultations, including access, technical care, continuity, and multiple attributes relevant to patient- centred care. | | Discrete choice experiment | | 1,193 patients from 6 family practices in England | | Access: number of days’ wait for an appointment.  Continuity: physician’s knowledge of the patient. | | Patients valued thoroughness of physical examination most highly, followed by seeing a physician who knew them well, seeing a physician with a friendly manner, having a reduction in waiting time of 1 day, and having flexibility in selecting appointment times.  Responses were influenced by the scenario in which the decision was made (minor physical problem vs urgent physical problem vs ambiguous physical or psychological problem) and by patients’ demographic characteristics. | | “What is important for patients?” | | **Preference for timely access over relational continuity and vice-versa was context/condition specific.** | |
| Haggerty et al. (2008) | Canada | To identify attributes of clinic organization and physician practice that predict accessibility, continuity, and coordination of care as experienced by patients. | | Questionnaire (Primary Care Assessment Tool) | | 2,725 patients from 100 primary health care clinics in Quebec, Canada | | Access: being able to obtain care promptly for sudden illness.  Continuity: having an ongoing relationship with a physician who knows the patient’s history. | | Offering scheduled visits in evenings improved relational continuity whereas the effect for weekends was not significant.  A walk in care and high-volume practice style was associated with lower relational continuity | | “Promoting access and continuity of care in general practice”. | | **Offering scheduled evening visits improved timely access and relational continuity.** | |
| Antoun et al. (2014) | Lebanon | To assess the priority of various aspects of the patient-primary care physician relationship in the decision to visit again that same physician | | Questionnaire | | A total of 400 community residents in Ras Beirut, Lebanon. | | Continuity: having adequate time for explanation and discussion of the problem and treatment.  Access:   - waiting time - helpfulness of the receptionist - availability of the physician’s private telephone number. | | Adequate time with the patient is the most important factor in the decision to visit again that same physician irrespective of socio-demographic or health status.  Short waiting time was found highly important for patient’s desire to return to the same clinic, and access to the physician by phone was considered highly important among women and those of lesser education status. | | “What is important for patients?” | | **Both timely access (short waiting time) and physical access (telephone access to GP) improved relational continuity** | |
| Locatelli et al. (2014) | US | To examine preferences for relational continuity and rapid accessibility for telephone care | | Mixed methods (structured telephone interviews + focus groups) | | Structured telephone interviews with 448 Veterans receiving primary care from Veterans Affairs facilities.  Seventeen focus groups with 123 Veterans care from Veterans Affairs facilities. | | Telephone interview questions included:   - Talking with a nurse from your own primary care team (who knows you) rather than a nurse from a call centre (who does not know you). - Talking with a nurse who you have had a previous primary care contact with (vs no previous contact). - Being able to talk to any nurse as soon as possible. - Receiving advice immediately from a nurse when you call versus being promised a call back by your primary care team within 1 hr. | | Overall, participants showed a strong preference for a familiar provider. This includes notions such as knowledge of the patient’s medical history, reduced need for “telling the whole story” to receive advice on a specific question or issue, and establishment of a long-term relationship between provider and patient.  Rapid accessibility was seen as optimal in certain situations, such as for urgent needs, but generally was not as strongly preferred as relational continuity. | | “Promoting access and continuity of care in general practice”. | | **Preference for relational and informational continuity over timely access over was context/condition specific.** | |
| Wetmore et al. (2014) | Canada | To determine patient satisfaction with care provided at a family medicine teaching clinic. | | Questionnaire | | 301 patients at the Victoria Family Medical Centre in London, Ontario receiving care from a multidisciplinary team that included multiple learners. | | Access: clinic hours and how patients felt about their ability to access the physicians at the clinic.  Continuity: wait time to see particular physician vs any physician. | | Patients were generally satisfied with the care provided. Points of dissatisfaction were related to wait times for appointments and continuity with patients’ usual doctors.  When patients booked appointments with any doctors, the median wait time was 2 working days; when patients requested specific doctors, the median wait time was increased by 1 day. | | “What is important for patients?” | | **Requesting to see specific GPs (relational continuity) reduced timely access.** | |
| Altin et al. (2015) | Germany | Explores if health literacy and patient-reported experiences regarding access to care and support in care-coordination in primary care organizations have an impact on patients satisfaction with the care received by their personal general practitioner. | | Questionnaire | | A random sample of 1125 German adults. | | Access: assessed using the item “When you call your regular doctor’s office with a medical concern during regular practice hours, how often do you get an answer that same day?”.  Care coordination: measured using the item “How often does your regular doctor or someone in your doctor’s practice help coordinate or arrange the care you receive from other doctors and places?”. | | Better accessibility of the primary care practice, frequent support in care coordination by the general practitioner as well as sufficient health literacy were independent predictors of a higher satisfaction with care received in the general practice. | | “What is important for patients?” | | **Timely access and management continuity led to higher satisfaction with care received by a personal general practitioner (relational continuity).** | |
| Panattoni et al. (2015) | US | Examine the relationships between a physicians’ clinical full-time equivalent, continuity of care, access to care, and patient satisfaction with the physician. | | Observational study + patient satisfaction survey | | Family medicine (n=104) and internal medicine (n=101) physicians in a multi-specialty group practice, along with their patient satisfaction survey responses (n=12,688). | | Access: the third next-available appointment which measures a primary care physician’s average number of days until the third next- available appointment.  Continuity:   - annual percentage of all primary care office visits made by a physician’s patients to their own physician. - annual percentage of a physician’s office visits that were spent seeing their own patients. | | Physician FTE was directly associated with better continuity of care received, better continuity of care provided, and better access to care, but worse patient satisfaction scores. | | “Practice-level factors that influence access and continuity of care”; “what is important for patients?” | | **GP FTE had a direct association with timely access and relational continuity.** | |
| Paddison et al. (2015) | UK | To describe the experiences of people with diabetes in primary care and examine how these experiences vary with increasing comorbidity. | | Questionnaire | | Using data from 906,578 responders to the 2012 General Practice Patient Survey (England), including 85,760 with self-reported diabetes | | Access:  - How easy is it to get through to someone at your GP surgery on the phone?  - Overall, how would you describe your experience of making an appointment?  Continuity:  - How often do you see or speak to the GP you prefer? | | Among people with diabetes, those with additional comorbid long- term conditions reported worse patient experiences, particularly for questions on access to appointments. The likelihood of being able to see the patient’s preferred doctor did not change substantially with increasing number of comorbid long-term conditions. | | “What is important for patients?” | | **Patients with co-morbidities reported worse experiences for physical and timely access but reported no difference in relational continuity.** | |
| Rosland et al. (2015) | US | Sought to examine whether differences in performance between part-time and full-time primary care providers (PCPs) were attenuated when moving from measures focusing on access to a patient’s usual PCP, to access to any clinic PCP, and from measures focusing on access to the usual PCP on the same day versus within 7 days. | | Retrospective analysis | | 110,454 primary care visits from 2 Veterans Health Administration clinics from 2010 to 2012. | | Access: percentage of all same-day requests that were completed by an encounter the same day or the next day with the   - patient’s usual (assigned) physician. - same-day other physician. - 2 to 7 Days usual physician.   Continuity: percentage of all completed in-person PC encounters in which the provider was the patient’s assigned physician. | | Patients of part-time PCPs had less same-day access with their usual PCP than patients of full-time PCPs.  Patients with same-day appointment requests were equally likely to obtain appointments with their usual PCP within 2 to 7 days, regardless of the number of PCP sessions per week.  Related to continuity, fulltime and part-time PCP patients reported similar rates of PCP knowledge of their medical history and specialist care | | “Practice-level factors that influence access and continuity of care”. | | **Patients of part-time primary care providers had reduced timely access to their usual GP (relational continuity)** | |
| Sav et al. (2015) | Australia | To investigate the healthcare priorities of consumers with chronic conditions and their carers, if there are differences between these two groups, and if priorities differ depending on geographical location. | | Nominal group technique | | 11 nominal groups were conducted; five groups consisted predominantly of consumers (n = 33 participants), two groups consisted predominantly of carers (n = 12 participants) and four were mixed groups, i.e. consumers, carers, and both (n = 26 participants). | | See relevant findings | | Appropriate and timely healthcare access was of paramount importance. Continuity and coordinated care, patient-centred care and affordability were equally the second most important healthcare priorities for all groups. | | “Promoting access and continuity of care in general practice”; “what is important for patients?” | | **Timely access, relational continuity and management continuity were high priorities for patients with chronic conditions.** | |
| Tarrant et al. (2015) | UK | To explore patients’ experiences of discontinuities in care and to gain insight into how gaps come to be bridged and why they might remain unresolved. | | Semi-structured interviews | | 50 patients, recruited from 15 general practices and one walk-in centre and community settings in Leicestershire, UK. | | See relevant findings | | Experiences of gaps in care were common, arising from failures in communication and coordination of care. Patients who experienced falling through gaps all described how they had had difficulties in establishing their eligibility for care and accessing appropriate services going forward. Bridging gaps required resources; relationship continuity was a valuable resource for preventing and repairing gaps in care. | | “What is important for patients?” | | **Better physical access and relational continuity helped prevent and repair gaps in care.** | |
| Hung et al. (2016) | US | Examined relationships between organizational culture and patient-centred outcomes in primary care. | | Questionnaire | | 357 physicians in 41 primary care departments. | | Access: waiting times for third-next-available “long” office visit appointments (30 to 45 minutes), typically used for routine physical examinations.  Continuity: percentage of primary care office visits made by a physician’s patient to that patient’s own physician.  Both measures were tracked by the organization at the physician level. | | This study found that different organizational culture was signiﬁcantly associated with various measures of patient access to care, continuity of care, and reported experiences with care delivery.  Compared with a “Group-oriented” culture, a “Rational” culture type was associated with longer appointment wait times, and both “Hierarchical” and “Developmental” culture types were associated with less care continuity, but better patient experiences with care. | | “Practice-level factors that influence access and continuity of care”. | | **Timely access and relational continuity were influenced by organizational culture type** | |
| Jego et al. (2016) | France | To analyse the views of general practitioners (GPs) about how they can provide care to homeless people (HP) and to explore which measures could influence their views. | | Mixed methods (semi-structured interviews + survey) | | 19 GPs involved in homeless people healthcare were recruited for phase 1 (qualitative).  For phase 2 (quantitative) 105 GPs who provide routine healthcare (‘standard’ GPs)  responded to the questionnaire  For phase 3 (qualitative), data were explored on 14 ‘standard’ GPs. | | See relevant findings | | GPs felt the most difficulties in the care giving for the homeless due to: social management, retrieving medical information, management of observance of homeless patient, loneliness in practice, time necessary for consultation and complexity of care management.  Maintaining a stable follow-up was a major condition for GPs to contribute effectively to the care of HP. Developing a medical and psychosocial approach with closer relation with social workers and enhancing the collaboration between tailored and non-tailored programmes were also other key answers. | | “Promoting access and continuity of care in general practice”. | | **Informational and management continuity were key to providing care for homeless people** | |
| Kenny et al. (2016) | Australia | To identify the factors considered important when choosing a GP and to explore the extent to which preferences are associated with individual health and demographic characteristics. | | Questionnaire | | Australian population sample (n = 2481) of adults aged 16 or more. | | Access:   - I can make appointments out of hours (i.e. night, weekends, holidays) - I can make an appointment online - The practice provides urgent care out of hours (i.e. night, weekends, holidays) - I can make a same day appointment - I can make an appointment to see a GP at a time of day that suits me - The practice offers alternatives to doctor face-to-face consultations (e.g. phone, email)   Continuity:   - I can choose whether to see the GP, GP assistant or practice nurse. - I can see the same GP each time and she or he knows my medical history. - I can see the GP of my choice. - The GP has easy access to my computerized medical records. | | The highest mean dimension scores were for care quality (mean 4.45, 95% CI 4.43–4.47) and cost (mean 4.13, 95% CI 4.10–4.15), where a higher score indicates greater importance for choosing a GP.  The individual items most frequently identified as important or extremely important (rated 4 or 5) were those making up the care quality dimension; the interpersonal care attributes (GP communication, information provision and length of consultation) were rated as important by 90– 92% of participants. | | “What is important for patients?”. | | **Timely access, physical access, and relational continuity were important factors for choosing a GP** | |
| Pineault et al. (2016) | Canada | To identify organizational characteristics associated with the size of primary health care practice and to determine the extent to which the influence of size on experience of care, preventive services, and unmet needs is mediated by these organizational characteristics. | | Questionnaire | | Population survey involved 9180 randomly selected adults, aged 18 or older.  Practice survey: included all primary health care practices (N = 606) in 2 regions. | | Access:   - If the physician who is responsible of your care is not available, can you see another physician? - How long does it take to see a physician by appointment? - How long does it usually take to get to this place? - The office hours are convenient. - It is easy to reach someone by telephone to make an appointment. - It is easy to talk to a physician or nurse by telephone.   Continuity:   - When you go to this place, do you see the same physician? - How long have you been going there? - The professionals know your medical history. - The professionals are aware of all your prescribed medications. - You can receive ongoing care for a chronic problem. | | Overall, the larger the size of a practice (measured by the number of physicians working in the practice regardless of the number of hours they spent in that practice), the higher the accessibility, but the lower the continuity. However, this effect is largely mediated by organizational characteristics, such as targeting regular patients rather than any individual who presents to the clinic or the broader population, being open in evenings and on weekends, and predominantly having scheduled visits as opposed to walk-in visits. | | “Practice-level factors that influence access and continuity of care”. | | **Greater number of physicians working in the practice had a direct relationship with timely access but indirect relationship with relational continuity.** | |
| Reid et al. (2016) | New Zealand | Examining how Māori describe their experiences of accessing and engaging with primary healthcare. | | Semi-structured interviews | | 42 purposively selected Māori adults | | See relevant findings | | The inability to access a usual GP was identified as a principal organisational barrier to receipt of optimal care for study participants. Access to a known GP seemed to be important for some participants as protective against the risk of discriminatory treatment. | | “What is important for patients?”. | | **Timely access to a known GP (relational continuity) improved patient engagement with primary care** | |
| Weir et al. (2016) | US | To describe a quality improvement approach implemented to maximize continuity of care and minimize delays for care | | A diverse quality improvement team from family medicine centre joined a breakthrough collaborative with other primary care practices focused on improving appointment access and continuity of care. | | Study took place at the University of North Carolina. (UNC) Family Medicine Center | | Continuity:   - Usual Provider Continuity (UPC) for each physician. - Usual Team Continuity (UTC) for each team of physicians.   Access: time to third available appointment (TTA) for each primary care physician. | | Average time to third available appointment for a routine physical improved from 22 days to 8 days.  Average usual provider continuity (UPC) across all primary care physicians in the practice improved from 54% to 68%. Among resident physicians,  UPC improved from 55% to 68%. These results were sustained over 5 years. | | “Promoting access and continuity of care in general practice”. | | **Interdisciplinary quality improvement interventions improved timely access and relational continuity.** | |
| Ehman et al. (2017) | US | To assess patient satisfaction and preferences regarding the trade-off between continuity of care and quick access in various scenarios. | | Questionnaire | | 770 patients at a family medicine clinic in Kasson, Minnesota | | Eight hypothetical scenarios including acute (sore throat, sinus infection, sprained ankle, stitches) and chronic issues (medication refill, high blood pressure follow-up, weight management, depression) were presented. For each scenario, respondents were asked to choose between seeing their primary physician within 1 week, seeing a care team physician within 3 days, and seeing any physician within 1 day to assess their preferences regarding the trade-off between continuity of care and quick access to care. | | All patients prefer continuity of care with their primary care physician (PCP) for chronic disease management and value quick access to care for acute problems. For acute visits, multimorbidity patients prefer to wait longer to see their PCP than healthy adults.  Patients who were not satisfied with their care team preferred to wait longer to see their PCP. Those not satisfied with their PCP choose to be seen sooner by their care team or any physician. | | “What is important for patients?”. | | **Preference for timely access over relational continuity and vice-versa was context/condition specific.** | |
| Lamanna et al. (2017) | Canada | To examine the role of a brief interdisciplinary intervention in supporting continuity of care for people experiencing homelessness in a large Canadian urban centre. | | Focus groups & semi-structured interviews | | Three focus groups were conducted with service providers and people with lived experience of homelessness, and 29 individual, semi- structured interviews were conducted with service users and other key informants. | | See relevant findings | | Findings suggest that brief interdisciplinary interventions can promote continuity of care by offering low-barrier access, timely and responsive service provision, including timely connection to long-term services and supports, appropriate individualised services and effective co-ordination of services. | | “Promoting access and continuity of care in general practice”. | | **Interdisciplinary interventions improved physical and timely access, as well as relational and management continuity** | |
| Abreu et al. (2018) | Brazil | To analyse service users’ perception of health care provided by the teams participating in the National Program for Primary Care Access and Quality Improvement (PMAQ-AB) in Brazil. | | Questionnaire | | 65,391 primary healthcare service users | | Access:   - presence of a physician at the health care centre or in activities in its site at all times at in which the health centre is open (yes or no). - the patient receives attention to solve any problem, regardless of whether or not they have an appointment (yes, yes/always, sometimes/no). - how long does the patient have to wait before being seen (0 to 15 minutes/15 to 30 minutes/more than 30 minutes).   Continuity:   - are you always seen by the same physician (yes/yes, sometimes/rarely/never). - do other health care team professionals visit your home (yes/yes, sometimes/yes, almost always/no). | | In relation to accessibility, the variable "presence of a physician at the health centre or in activities in its territories at all times in the health centre is operational” was associated with user perception as being very good/good. As regards "how long do you have to wait before being seen", it was found that in the case of waiting between 1 and 15 minutes, and between 16 and 30 minutes, the degree of association with the assessment of care provision classified as very good/good was similar.  The longitudinal variable "are you always seen by the same physician" had a positive association with very good/good user evaluation when care is always provided by the same physician and in cases in which care is provided several times by the same physician (OR 2.60; 95% CI 2.14; 3.29). | | “What is important for patients?”. | | **There was a positive association between timely access; physical access; relational continuity and good user evaluation of primary healthcare.** | |
| Droz et al. (2019) | Switzerland | To describe patients’ most important values regarding family medicine and to assess their associations with socio-demographics factors in a fee-for-services health system. | | Questionnaire | | A sample of two hundred patients randomly drawn from national lists of GPs associations | | Access:   - The practice has extensive opening hours. - I can get an appointment easily at this practice. - I know how to get evening, night and weekend services. - The practice is close to where I live or work. - I have a short waiting time on the phone when I call this practice. - The doctor does not give me the feeling to be under time pressure. - The doctor offers me his/her telephone or email to contact for further questions. - I will keep my appointment.   Continuity:   - The doctor has my medical records at hand. - The doctor knows important information about my medical background and health issues. - The doctor knows about my living situation. - The doctor has prepared for the consultation by reading my medical notes. - The doctor gives me instructions on what to do when things go wrong. - I do not need to tell a receptionist or nurse details about my health problem. - I inform the doctor how the treatment works out. - I can see another doctor if I think it is necessary. | | The most important value of patients regarding family medicine are communication, continuity and coordination of care. Continuity and coordination of care are systematically valued as more important by patients with a chronic disease. | | “What is important for patients?”. | | **Factors relative to relational and informational continuity were perceived as more important than timely and physical access.** | |
| Forman et al. (2019) | US | To understand how academic primary care clinics provide continuity to patients requesting same-day access and identify factors that may affect site-level success. | | Semi-structured interviews | | 17 physicians and 2 nurse practitioners from 19 Veterans Health Administration academically- affiliated medical centres | | See relevant findings | | Most sites took a team-based approach to ensure continuity and provide coverage for same-day access, notably using nurse practitioners, PAs, and registered nurses in their coverage algorithms. They reported several adaptations that increased multiple types of continuity for walk-in patients, urgent care between in-person visits, and follow-up care. | | “Promoting access and continuity of care in general practice”. | | **A team-based approach improved timely access and informational continuity** | |
| Norwood et al. (2019) | Portugal | To examine patients’ experiences of and preferences for primary care in Portugal and to explore their experience of the recent reforms. | | Focus groups | | Eight focus groups with patients from eight local institutions in the city of Braga, Portugal. | | See relevant findings | | Participants’ satisfaction/ dissatisfaction with primary care was strongly associated with interpersonal relations and communication with doctors. Participants valued continuity of care, but felt the levels of responsiveness, flexibility and coordination in the current system were still unsatisfactory. Access and waiting times were seen as challenging and led participants to seek care from emergency departments and private doctors. | | “System-level factors that influence access and continuity of care”; “promoting access and continuity of care in general practice”; “what is important for patients?”. | | **Patients wanted better choice and timely access an with their personal GP.** | |
| Oliver et al. (2019) | Canada | Examined trade-offs that patients may consider during appointment bookings for six different clinical scenarios across a number of key access and continuity attributes | | Discrete choice experiment | | Convenience sample of 430 patients of family medicine clinics aged 18 and older. | | Access:   - I can book an appointment: on the internet, right now; over the phone, and wait less than 1min; over the phone, and wait 1 to 10 mins until it is answered. - The appointment time is: Exactly the time of day I want; Not exactly the time of day I want, but okay; Not a good time at all. - I will spend <15; 15-30; >30 minutes in the waiting room.   Continuity:   - I get to see a healthcare provider: the same day; in 1-14 days; in more than 14 days. - I will see a healthcare provider who knows me: well/not very well/not at all. - The healthcare provider is a: family doctor; training doctor (resident); nurse/nurse practitioner. | | Patients rated appointment wait time as the most highly valued attribute, followed by position of provider, then familiarity with the provider. Patients showed a significant preference for their own physician for booking of routine annual check-ups and other logical preferences across attributes overall and by clinical scenario. | | “What is important for patients?”. | | **Preference for timely access over relational continuity and vice-versa was context/condition specific.** | |
| Cook et al. (2020) | Canada | To explore the relation between a change in access to a primary care physician and continuity of care | | Retrospective cohort study among physicians in a primary care network in southwest Alberta who measured access consistently between 2009 and 2016. | | Analysed data from 190 primary care physicians | | Access: the third next available appointment (TNA) metric, which is the delay patients experience accessing providers for a scheduled short appointment (typically 10–15 min in duration, for routine primary care encounters). A TNA value of 0 indicates that a patient could have a same-day appointment, whereas a TNA value of 14 indicates that a= patient would experience a 2-week delay to get an appointment. | | Physicians with improved access increased provider continuity by 6.8% per year, reduced discontinuity by 2.1% per year, and decreased emergency department encounters by 78 visits per 1000 patients per year compared to physicians with stable access. Physicians with worsening access had a 6.2% decrease in provider continuity and an increased number of emergency department encounters (64 visits per 1000 panelled patients per year) compared to physicians with stable access. | | “What is important for patients?”. | | **Timely access had a direct relationship with relational continuity** | |
| Forbes et al. (2020) | UK | To explore whether increasing the size of the practice population and working collaboratively are linked to changes in continuity of care or access to care. | | Observational study which used data on patient experience, practice size, and collaborative working from the English GP Patient Survey, NHS Digital, and from a previous study. | | Analyses were carried out on the 7089 practices with data available on NHS Digital about practice size for 2013 and 2018, as well as data for both the 2013 and 2018 GP Patient Surveys. | | Access:   - How satisfied are you with the hours that your GP surgery is open?’ - Generally, how easy is it to get through to someone at your GP surgery on the phone? - Were you able to get an appointment to see or speak to someone, the last time you wanted to see or speak to a GP or nurse at your GP surgery?   Continuity:   - How often do you see or speak to the GP you prefer? applicable to responders who replied ‘yes’ to the question: “Is there a GP you usually prefer to see or speak to?”. | | Practices that had grown in population size had a greater fall in continuity of care (by 6.6%, 95% confidence interval = 4.3% to 8.9%), than practices that had roughly stayed the same size, after controlling for other factors.  Differences in falls in access to care were smaller (4.3% difference for being able to get through easily on the telephone; 1.5% for being able to get an appointment; 0.9% in satisfaction with opening hours), but were statistically significant.  Practices collaborating closely with others had marginally worse continuity of care than those not working in collaboration, and no differences in access. | | “Practice-level factors that influence access and continuity of care”. | | **Increasing practice population size reduced relational continuity, but had a negligible effect on physical and timely access.** | |
| Krzton-Krolewiecka et al. (2020) | Poland | To compare the expectations and experiences of patients of primary care in Poland. | | Questionnaire | | A nationally representative sample of 2218 patients in Poland | | Access:   - Easy appointment scheduling. - Short waiting time during the phone call to the practice. - Patient awareness about out of hours medical services. - Nearby location of the practice. - Extensive opening hours in the practice.   Continuity:   - Doctor’s direct access to patient’s medical record. - Doctor’s familiarity with patient’s living conditions. - Doctor’s familiarity with patient’s medical background and health issues. | | Patients’ expectations were fulfilled in all study areas: accessibility, continuity, quality of care, and equity. Accessibility, continuity, quality of care, and equity in primary care were important for respondents with accessibility being the most crucial area, and continuity the least important. | | “What is important for patients?”. | | **Factors relative to timely and physical access were perceived to be of higher importance than those of relational and informational continuity** | |
| Kuipers et al. (2020) | Netherlands | To identify views of patients with multi-morbidity on the relative importance of patient-centred care (PCC) aspects in a Dutch primary care setting. | | Q-methodology | | Interviews were conducted with 16 patients in the Netherlands with multi-morbidity | | See relevant findings | | Three viewpoints regarding these aspects were identified. Patients with viewpoint 1 are the prepared proactive patients who seem to be well-off and want to be in charge of their own care. To do so, they seek medical information and prefer to be supported by a strongly coordinated multidisciplinary team of healthcare professionals.  Patients with viewpoint 2 are everyday patients who visit GPs, and are in need of well-coordinated, respectful, and supportive care.  Patients with viewpoint 3 are vulnerable patients who are less resourceful in terms of communication skills and finances, and are thus in need of accessible care and professionals’ lead taking while treating them with dignity and respect. | | “What is important for patients?”. | | **Proactive patients who want to be in charge of their own care have a strong preference for management continuity.**  **Patients who visit GPs daily highly value relational continuity.**  **More “vulnerable patients” place high emphasis on both access and relational continuity.** | |
| Murphy et al. (2020) | UK | To examine the attributes of GPs that patients with long-term conditions value most, and which attributes patients believe are facilitated by relational continuity. | | Semi-structured interviews | | 25 patients with long-term conditions purposefully sampled from five NHS sites in Bristol including three GP practices | | See relevant findings | | Key GP attributes that patients prioritised were categorised into attitude- and approach-related attributes, and knowledge- and competence-related attributes. Patients focused mostly on the importance of attitude and approach, with listening, trust and respect, and taking responsibility and action being the three most important | | “Practice-level factors that influence access and continuity of care”. | | **Patients with long-term conditions valued relational continuity and informational continuity but did not mention any attributes of access to influence relational continuity.** | |
| De Foo et al. (2021) | Singapore | To map features of PCN to Starfield’s “4Cs” framework (comprehensiveness, first contact access, coordination and continuity) on qualitative data to provide deep understanding of how PCN empowers GPs to manage patients with chronic conditions through the attainment of the “4Cs”. | | Semi-structured interviews | | 30 GPs enrolled in a PCN were purposefully selected | | See relevant findings | | Provision of ancillary services, manpower, a chronic disease registry and extended operating hours of GP practices demonstrated PCN’s empowering features that fulfil the “4Cs”. On the contrary, operational challenges such as the lack of an integrated electronic medical record and disproportionate GP payment structures limit PCNs from maximising the “4Cs”. | | “Promoting access and continuity of care in general practice”. | | **GPs operating long opening hours (evenings and weekends) improved timely access, particularly for working people.**  **Having a disease registry for patients with chronic conditions promoted informational continuity.** | |
| Kuipers et al. (2021) | Netherlands | To describe how primary care could be improved for patients with multimorbidity by evaluating a program designed to improve patient-centred care (PCC) delivery to these patients in general practitioners’ (GPs’) practices in the Netherlands | Mixed methods (semi-structured interviews + questionnaire) | | Semi-structured interviews were conducted with nine general practitioners and nurse practitioners from seven primary care practices in Noord-Brabant, the Netherlands.  The longitudinal survey was conducted with 138 patients with multimorbidity from these practices. | | *Questionnaire*  Access:   - The building is accessible to all clients. - Clear directions are provided to and inside the building. - It is easy to schedule an appointment. - Waiting times for an appointment are acceptable. - Language is not a barrier for access to care.   Continuity:   - When a patient is transferred to another ward, relevant patient information is transferred as well. - Patients who are transferred are well-informed about where they are going, what care they will receive and who will be their contact person. - Patients get expert advice about care and support at home after discharge. | | Themes necessary for PCC improvement according to healthcare professionals involved: information and education; improving access to care, coordination of care within GP practice; continuity of care across settings, emotional support, family and friends. | | “Promoting access and continuity of care in general practice”. | | **Improving timely access, physical access, and management continuity across settings were perceived as important pillars of patient-centred care for patients with multi-morbidities in primary care settings.** | |  |
| Norwood et al. (2021) | Portugal | To evaluate public preferences for relational continuity of care alongside other attributes of Primary Care services in Portugal | | Discrete choice experiment | | 527 members of the public from municipality of Braga-Portugal | | Access: the GP practice is easy to reach, with good public transport and close by parking available (yes/no).  Continuity: who is the appointment with (the usual GP/ not the usual GP). | | Participants’ satisfaction/ dissatisfaction with primary care was strongly associated with interpersonal relations and communication with doctors. Participants valued continuity of care, but felt the levels of responsiveness, flexibility and coordination in the current system were still unsatisfactory.  Access and waiting times were seen as challenging and led participants to seek PC from emergency departments and private doctors. | | “System-level factors that influence access and continuity of care”; “promoting access and continuity of care in general practice”. | | **Patients prefer relational continuity along with timely and choice** | |
| Homburg et al. (2022) | Netherlands | To assess the experiences of patients in primary care during the COVID-19 pandemic. | | Semi-structured interviews | | Twenty-eight patients were interviewed (13 men and 15 women, aged 27–91 years). | | See relevant findings | | Multiple patients reported appreciating that they had more time scheduled for GP appointments during the COVID-19 pandemic, which they thought had improved the quality of care.  Patients experienced practices as being quieter with few patients around and, given that this reduced waiting times and increased consultation times for them, they evaluated this positively.  Responses indicated that some practices offered specific office hours for care related to COVID-19 infection, with this sometimes provided at different locations and by practitioners other than their GP. This led to a decreased quality of relationship with their own GP, and with that, to a decrease in continuity of in-person care. | | “Practice-level factors that influence access and continuity of care”; “what is important for patients?”. | | **In some instances, COVID-19 restrictions led to improved timely access for some patients.**  **Offering** specific **office hours for care related to COVID-19 infection at different GP practices decreased the relational continuity of care and in turn the perceived quality of care.** | |
| Lautamatti et al. (2022) | Finland | To investigate whether a named and assigned GP representing continuity of care is associated with the use of primary and hospital health care services and to create knowledge on the state of continuity of care in a changing health care system in Finland. | | Survey based on a random Finnish working age population sample of 64,797 individuals drawn in 1998 and follow-up surveys in 2003 and 2012. | | The response rate in 1998 was 40% (n = 25,898). Continuity of care was derived from the 2003 and 2012 data sets, other variables from the 2012 survey (n = 11,924). | | Access: how many times the participant had visited a doctor at a health centre during the last year: 0, 1, 2‒4 and 5 or more times. The response was either zero or one visit to the services and > 1 as two or more visits.  Continuity: “do you have an assigned and named GP at your local health centre?” (“yes” or “no” indicated the existence or lack of named GP). | | A named and assigned GP was independently and significantly associated with more frequent use of primary and hospital care in the adjusted logistic regression analysis (ORs 1.53 (95% CI 1.35–1.72) and 1.19 (95% CI 1.08–1.32), p < 0.001). | | “Practice-level factors that influence access and continuity of care”. | | **Relational continuity had a direct relationship with physical access** | |
| Marshall et al. (2022) | Canada | Attachment to a regular primary healthcare (PHC) provider, a key to healthcare access, has seen a decline in some jurisdictions. This study explored the consequences of unattachment from a patient perspective | | Semi-structured interviews | | Nine unattached patients | | See relevant findings | | Seven of the nine participants felt that they had diagnoses that were either incorrect, delayed, or missed altogether due to the discontinuity of PHC and the absence of having a complete medical record available for interim care without a regular PHC provider.  Walk-in clinics were the primary source of PHC for participants while unattached. While some were able to use the same walk-in clinic repeatedly to approximate attachment, most found them to be frustrating due to wait times, feeling rushed in the appointment, reluctance of walk-in doctors to order tests, write referrals, or write certain prescriptions, having to be the reporter of their own medical history, and the perceived incompetence of some walk-in physicians. | | “System-level factors that influence access and continuity of care”; “what is important for patients?”. | | **Lack of access to relational and informational continuity negatively impacted patient care. Access to walk-in clinics reduced the (quality) of relational continuity.** | |
| Slater et al. (2022) | UK | A Scottish GP practice proposed an improvement intervention, shorter pre-bookable ‘review’ appointments, to increase appointment capacity and meet their patients’ demand for appointments.  The aim of this project was to establish if patient demand for appointments was being met, with a target of 95% of patients receiving an ‘on the day’ appointment when they phone for one between 08:00 and 09:00. | | The intervention proposed was to include an allocated 30-minute slot per morning and afternoon session for review appointments, where GPs and advanced nurse practitioners (ANPs) would see four patients (with 7.5-minute appointments) instead of the two patients they saw in this time previously. | | Quantitative data were collected to assess the number of patients phoning between 08:00 and 09:00 who received an ‘on the day’ appointment with the use of a tally sheet, with data collected every morning between 08:00 and 09:00 for a 7-week period, 3 months post-intervention. The percentage of patients receiving an appointment when they phoned for one between 08:00 and 09:00 was calculated. This information represents the outcome measure of the project—measurement of how much the system impacts patients. | | Access: receiving a same day appointment when patients phone for one between 08:00 and 09:00am.  Continuity: staff can pre-book these review appointments for patients, guaranteeing they will see the same GP/ANP for both appointments. | | During a 7-week period, 3 months post-intervention, a median of 93% of patients received an 'on the day' appointment when they phoned for one between 08:00 and 09:00. The number of appointments available increased by 43%.  The implementation of review appointments increased continuity of care, increased the number of patients able to be seen and decreased the number of frustrated patients the administrative staff deal with. Patients also prefer being able to book in advance and to be able to see the same GP/ANP.  Barriers to the system, as identified by administrative staff, include: the lack of later appointments for patients who work; the timings of emergency appointments; the increased rate of DNAs (‘did not attend’) associated with appointments that are pre-booked (online and review); and the inability of some patients to phone at 08:00 for an appointment. | | “Practice-level factors that influence access and continuity of care”; “promoting access and continuity of care in general practice”; “What is important for patients?”. | | **Shorter duration pre-bookable review appointments guaranteeing patients will see the same GP increased timely and physical access and relational continuity of care for some patients.** | |
| Smithman et al. (2022) | Canada | To evaluate changes in access to and continuity of primary care associated with attachment to a family physician through Quebec’s centralized waiting lists for unattached patients. | | Observational longitudinal population cohort study of patients attached to a family physician through centralized waiting lists for unattached patients in Quebec between January 1st 2010 and August 31st 2015. | | Medical services billing data was to compare patients’ utilization of primary care pre- and post-attachment. All patients who had a billing code for attachment to a family physician through Quebec’s centralized waiting lists between January 1st, 2012 and August 31st, 2014. | | Access: number of visits to a family physician in a 12-month period.  Continuity: Concentration of Care Index – the number of visits to each individual physician divided by the total number of visits the patient had overall. | | With regard to access, results show a statistically significant and substantial increase in the number of primary care visits to a family physician. Compared to the reference year, the number of primary care visits doubled in the first year after attachment and was 29% higher in the second year.  As measured by the Bice-Boxerman Concentration of Care Index, results showed a post-attachment increase in the odds of having all primary care visits concentrated versus all visits being dispersed. | | “System-level factors that influence access and continuity of care”; “promoting access and continuity of care in general practice”. | | **Formal policies promoting attachment to a GP may help improve physical access and relational continuity** | |
| Voorhees et al. (2022) | UK | To fill the gap between the complex understanding of primary care access in the literature and the narrow definition of access assumed in UK policies. | | Multiple qualitative methods (interviews, focus groups, and observation) with service users (patients, carers, and voluntary sector roles) and service providers (GPs, practice managers, receptionists, commissioners, and other relevant NHS roles). | | 19 semi-structured interviews (9 service users, 10 service providers).  7 focus groups (6 focus groups with service users [n=30] participants), 1 focus group with service providers [n=5])  71 hours of observation (surgeries, meetings and events) | | See relevant findings | | In small practices receptionists often provided significant continuity for the patients, knowing them and their circumstances well.  Participants recognised that continuity was important across the clinical team. While patients requested clinicians who were known to them, many practice appointment systems were unable to accommodate this, leading to conflicts.  Continuity can be an important component of good access. The human skills of all members of the practice team are important in determining whether patients feel ‘known’ and therefore understood. Moreover, the current context of general practice with high workload and shortages of staff requires patients to also be adaptable and to show understanding of the pressures being experienced by staff. | | “Practice-level factors that influence access and continuity of care”; “what is important for patients?“; “promoting access and continuity of care in general practice”. | | **Improving timely and physical access could improve relational continuity, providing more opportunity to achieve that human fit between staff and patients.** | |
| Wang et al. (2022) | China | To profile high-quality primary health care from the perspective of the Chinese public. | | Semi-structured interviews | | 58 interviews conducted in 6 provinces (Henan, Shandong, Zhejiang, Shaanxi, Shanxi, and Heilongjiang) | | See relevant findings | | Patients’ perspective of good access involved GP surgery being located near their home; short waiting time for an appointment; and extended opening hours.  When “good” access was achieved, patients expected to be seen by the same GP each time who knew their medical history and could easily access it**.** Patients also expect their GP to follow-up with them post recovery. | | “What is important for patients?”. | | **Patients prefer physical and timely access to the same GP (relational continuity).**  **Patients valued informational continuity as it enabled their GP to be up-to date with their condition.** | |
| Del Grande et al. (2023) | Canada | To identify the top priorities of patients and clinicians for the organization of primary cardiovascular care in Quebec | | Delphi study | | Thirty panellists completed the study (9 clinic patients, 7 patient partners and 14 clinicians. | | Access:   - Being able to get an appointment with your family doctor on short notice. - Being able to reach a healthcare professional within 24-48 hours in the event of a problem, either on site, by phone, video conference or email. - Having the option to get longer consultations. - Having access to all clinic services in the evening and on weekends. - Having access to all clinic services in French or English. - Being seen on time for an appointment with little or no delay. - Free parking near the clinic.   Continuity:   - Having a single, common medical record between all healthcare providers. - Ensuring consistency in the professionals who follow the patient (same doctor, same nurse, etc.). | | Being able to reach a healthcare professional from the clinic quickly in the event of a problem, ensuring relational and informational continuity of care, as well as keeping professional skills up to date were amongst the top priorities shared by patients and clinicians. | | “What is important for patients?”. | | **Timely access, relational and informational continuity were top priorities in primary care for patients with cardiovascular disease** | |
